# Steep, coincident, and concordant clines in mitochondrial and nuclear‐encoded genes in a hybrid zone between subspecies of Atlantic killifish, *Fundulus heteroclitus*

**DOI:** 10.1002/ece3.2324

**Published:** 2016-07-22

**Authors:** Jessica L. McKenzie, Rashpal S. Dhillon, Patricia M. Schulte

**Affiliations:** ^1^ Department of ZoologyUniversity of British ColumbiaVancouverBritish ColumbiaCanada; ^2^ Centre for Aquaculture and Environmental ResearchFisheries and Oceans CanadaWest VancouverBritish ColumbiaCanada; ^3^ Present address: Department of Biomolecular ChemistryEpigenetics ThemeWisconsin Institute for DiscoveryUniversity of WisconsinMadisonWisconsin

**Keywords:** Bimodal, mtDNA, mummichog, selection, SNPs

## Abstract

Steep genetic clines resulting from recent secondary contact between previously isolated taxa can either gradually erode over time or be stabilized by factors such as ecological selection or selection against hybrids. We used patterns of variation in 30 nuclear and two mitochondrial SNPs to examine the factors that could be involved in stabilizing clines across a hybrid zone between two subspecies of the Atlantic killifish, *Fundulus heteroclitus*. Increased heterozygote deficit and cytonuclear disequilibrium in populations near the center of the mtDNA cline suggest that some form of reproductive isolation such as assortative mating or selection against hybrids may be acting in this hybrid zone. However, only a small number of loci exhibited these signatures, suggesting locus‐specific, rather than genomewide, factors. Fourteen of the 32 loci surveyed had cline widths inconsistent with neutral expectations, with two SNPs in the mitochondrial genome exhibiting the steepest clines. Seven of the 12 putatively non‐neutral nuclear clines were for SNPs in genes related to oxidative metabolism. Among these putatively non‐neutral nuclear clines, SNPs in two nuclear‐encoded mitochondrial genes (SLC25A3 and HDDC2), as well as SNPs in the myoglobin, 40S ribosomal protein S17, and actin‐binding LIM protein genes, had clines that were coincident and concordant with the mitochondrial clines. When hybrid index was calculated using this subset of loci, the frequency distribution of hybrid indices for a population located at the mtDNA cline center was non‐unimodal, suggesting selection against advanced‐generation hybrids, possibly due to effects on processes involved in oxidative metabolism.

## Introduction

When two previously isolated taxa come into contact, they may transiently form a hybrid zone, but in the absence of intrinsic or extrinsic mechanisms that maintain reproductive isolation the hybrid zone will gradually degrade as the taxa merge due to interbreeding (Barton and Hewitt 1985, 1989). Endogenous selection due to incompatibilities that cause hybrid inviability, sterility, or decreased fitness can maintain a hybrid zone, irrespective of environmental factors (Dobzhansky 1940; Moore and Price 1993). Alternatively or in addition, exogenous environmental selection can stabilize hybrid zones along an environmental gradient if parental taxa and/or offspring are differentially suited to divergent habitats (Moore and Price 1993). Endogenous and exogenous selection can interact, and even become coupled in a positive feedback loop such that local adaptation accounts for the position of the zone, while endogenous selection accounts for its persistence (Bierne et al. 2011).

Examining clines in allele frequencies across a hybrid zone can be a powerful way to detect genes that are responding to endogenous or exogenous selection, as these processes are likely to result in clines in selected loci that are steeper than the neutral clines (Vasemägi 2006). However, the power to detect these putatively selected loci can be low, particularly when many loci display clinal patterns as a result of recent secondary contact between moderately divergent taxa (Strand et al. 2012). In addition, even if outlier clines are detected, the conclusion that selection has shaped this pattern can only be a hypothesis because neutral processes such as allele surfing has the potential to result in similar patterns (Excoffier and Ray 2008). Additional insight can be gained by taking a multilocus approach (Harrison and Bogdanowicz 1997) and examining the frequency distribution of genotypic classes within a hybrid zone. Using this approach, hybrid zones can be classified along the continuum from complete admixture to complete reproductive isolation (Jiggins and Mallet 2000). With complete admixture, the frequency of genotypic classes approximates a normal distribution, and the zone is characterized as unimodal. In this case, hybrid types predominate, indicative of weak or absent reproductive isolation between the parental taxa. With near‐complete reproductive isolation between the two parental taxa, the frequency distribution of genotypic classes will have a bimodal distribution, in which most individuals are similar to one of the two parental types, and hybrids are present only at a low frequency if at all. A flat hybrid zone is intermediate between the other two types, and contains approximately even mixtures of parental and hybrid individuals. This pattern can be indicative of various combinations of forces, or may be the result of a relatively recently formed hybrid zone that is in transition between the initial bimodal state and the final state of Hardy–Weinberg equilibrium. Thus, determining the pattern of genetic variation within a hybrid zone is an important step in identifying the factors that may maintain the zone.

*Fundulus heteroclitus,* or the Atlantic killifish, is an abundant topminnow found in estuarine marshes along the east coast of North America, ranging from Newfoundland to northern Florida (Hardy 1978). Currently, two subspecies are recognized: *Fundulus heteroclitus heteroclitus* occupies the southern end of the distribution while *Fundulus heteroclitus macrolepidotus* is found in the northern end of the distribution. Patterns of genetic, embryological, and morphological differences between the subspecies support a model of secondary intergradation in which two previously isolated forms diverged in allopatry and then came back into contact. The center of this hybrid zone is thought to be located in northern New Jersey (Morin and Able 1983; Able and Felley 1986; Gonzáles‐Villasenor and Powers 1990; Ropson et al.^1990^; Adams et al. 2006), which coincides with the southern‐most extent of the Pleistocene ice sheet (Mickelson et al. 1983). This history of secondary contact has resulted in the formation of geographical clines at many loci in this species (Strand et al. 2012).

Previous work has shown that latitudinal genetic clines in *F. heteroclitus* vary substantially in steepness (Powers et al. 1986; Strand et al. 2012), with the steepest clines so far detected being for single nucleotide polymorphisms (SNPs) in the mitochondrial genome and for allozymes of a nuclear‐encoded, but mitochondrially localized isozyme of malate dehydrogenase (MDH) (Strand et al. 2012). However, it is unclear whether these steep clines are the result of selection on these loci or occur as a result of neutral demographic processes such as secondary contact. Recent theoretical work (Irwin 2012) indicates that for uniparentally inherited genomes such as the mitochondrial genome, even fairly weak selection can lead to the formation of sharp phylogeographic breaks in mitochondrial genotype if dispersal is moderate and population size is large. *Fundulus heteroclitus* fulfills these conditions, as these fish are present in extremely large numbers in their estuarine marsh habitats, with high effective population size (on the order of 10^5^ individuals; Adams et al. 2006), and dispersal between marshes along the coast is likely to be relatively limited (Fritz et al. 1975; Lotrich 1975). In addition, extensive previous work on clines in an isozyme of lactate dehydrogenase (*Ldh‐B*) has been interpreted as evidence that exogenous environmental selection is acting in this hybrid zone. There is a moderately steep cline in *Ldh‐B* allele frequency, and amino acid differences at this locus have been shown to differ functionally in ways that are consistent with selection in response to environmental temperature variation (Powers et al. 1979; DiMichele and Powers 1982a,b; Place and Powers 1984; Paynter et al. 1991; DiMichele and Westerman 1997). Similarly, a variety of analyses suggest that variation in gene expression, particularly at metabolic genes, may also be under thermal selection in this species (Schulte et al. 1997, 2000; Whitehead and Crawford 2006).

Using microsatellite markers, we have shown that the majority of clines in these markers were coincident with (i.e., shared the same center as) a cline in allele frequency of a SNP in the mitochondrial D‐loop and that the widths of these clines were narrower than what would be predicted in the absence of selection. Furthermore, individuals from a marsh located at the center of this hybrid zone exhibited a bimodal pattern of hybrid indices, again implicating selective forces in the maintenance of this zone (McKenzie et al. 2015). To extend these analyses and provide additional insight into whether non‐neutral processes may be involved in maintaining patterns of genetic variation in *F. heteroclitus,* we examined clines in 30 nuclear SNPs that are fixed or nearly fixed in populations from the extremes of the subspecies' distributions (identified based on Strand et al. 2012). We focused our analysis on populations from within the putative contact zone between the subspecies. We computed hybrid index based both on the complete set of SNPs and on a subset of loci that exhibited coincident and concordant clines along the coast. These analyses allowed us to examine whether processes such as selection against hybrids or assortative mating could be involved in maintaining this hybrid zone. As many these SNPs are located in protein‐coding genes of known function, this approach provides the opportunity to develop hypotheses about potential targets of exogenous and/or endogenous selection, which is not possible when using data from anonymous microsatellite markers.

## Materials and Methods

### Fish collection

**Figure 1:**
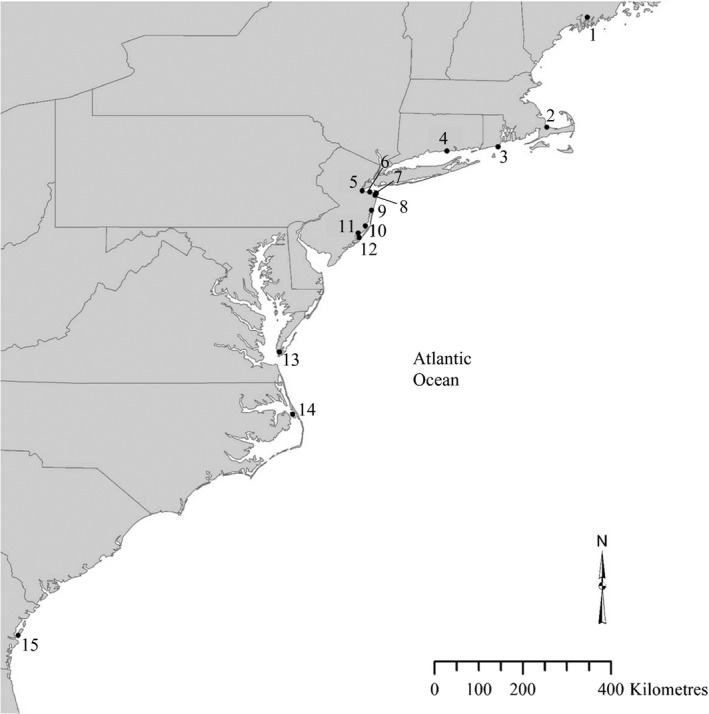
Map of sampling locations. Numbers correspond to the locations given in Table 1.

*Fundulus heteroclitus* were collected in May–June of 2008 from six locations along the Atlantic coast of North America, focusing on the putative contact zone between the two subspecies (Table 1; Fig. 1), using minnow traps with trap set time ranging from 2 to 6 h as reported in McKenzie et al. (2015). Fish length was recorded, and a fin clip was taken from each individual and preserved in 95% ethanol. DNA was extracted from these samples using a Qiagen DNeasy^®^ Blood and Tissue Kit (Qiagen Inc., Valencia, CA). SNP genotypes for individuals from an additional eight locations outside of the putative contact zone (Table 1; Fig. 1) have been previously published (Williams et al. 2010).

**Table 1:** Location of collection sites and sample sizes

Location	Distance to Sapelo Island, GA (km)	Collection	Latitude (°N)	Longitude (°W)	*n*
1. Wiscasset, ME	1968.44	Williams et al. (2010)	44.00289	−69.66558	30
2. Sandwich, MA	1691.84	Williams et al. (2010)	41.75896	−70.49393	20
3. Point Judith, RI	1591.72	Williams et al. (2010)	41.36538	−71.48672	21
4. Clinton, CT	1504.04	Williams et al. (2010)	41.27872	−72.52761	21
5. Cheesequake, NJ	1322.31	Current study	40.463420	−74.258888	39
6. Belford, NJ	1308.90	Current study	40.440968	−74.103963	50
7. Sandy Hook, NJ	1297.07	Current study	40.412904	−73.969487	50
8. Navesink, NJ	1292.51	Current study	40.376297	−73.993993	13
9. Metedeconk, NJ	1257.39	Current study	40.065616	−74.065320	49
10. Laurel, NJ	1220.73	Current study	39.750349	−74.192734	49
11. Tuckerton, NJ	1200.06	Williams et al. (2010)	39.60317	−74.34015	20
12. RUMFS, NJ	1189.90	Current study	39.512905	−74.320450	50
13. Magotha, VA	890.28	Williams et al. (2010)	37.18014	−75.94882	20
14. Manteo, NC	744.30	Williams et al. (2010)	35.90823	−75.67573	20
15. Sapelo Island, GA	0	Williams et al. (2010)	31.39745	−81.27871	33

### Single nucleotide polymorphism (SNP) genotyping

Fish were genotyped at 30 nuclear SNPs and two mitochondrial SNPs selected from an existing panel of 458 genomewide SNPs from *F. heteroclitus* (Williams et al. 2010; Strand et al. 2012). To develop this 32 SNP panel, we used JMP Genomics 3.2 for SAS 9.1.3 to conduct SNP case–control trait association tests to identify SNPs that differed significantly in genotype frequency between the extreme northern and southern populations surveyed by Williams et al. (2010) (Wiscassett, ME, and Sapelo Island, GA; 20 individuals at each site). We further narrowed this set by selecting the SNPs that were closest to fixation in the more genetically diverse southern population. We made this selection to increase the fraction of SNPs in the panel that were diagnostic or semi‐diagnostic for “northern” or “southern” genomic contributions in hybrid individuals. A total of 41 SNPs met our selection criteria, and these sequences were used by the McGill University and Génome Québec Innovation Centre to design a custom genotyping assay using Sequenom^®^ iPLEX^®^Gold Genotyping Technology (Sequenom, Inc., San Diego, CA). Nine of these SNPs failed to meet the quality control standards of the assay, and exhibited poor amplification, detection of multiple loci, or unreliable identification of hybrid individuals, and thus were dropped from the final SNP panel, which consisted only of those SNPs that could be scored with high accuracy (Table S1).

This Sequenom assay was used to genotype the 250 individuals collected for this study. Genotypes at these SNPs for an additional 235 individuals, also obtained using Sequenome, were taken from Williams et al. (2010) resulting in a total of 485 individuals across 15 locations available for population genetic analysis (Table 1).

To confirm the identity of the SNPs, we used BLASTN to compare the SNP flanking sequences to a draft version of the *F. heteroclitus* genome (www.fundulus.org). All SNP flanking sequences localized to only a single region of the genome, suggesting that our assay is robust against false positives due to detection of paralogs. The identities of the SNP‐containing loci were taken from the genome annotation tracks and were also manually curated by BLAST comparison against the NCBI nucleotide database (Table 2). The location of each SNP (i.e., coding, untranslated, intron, or intergenic) and its effect on amino acid sequence was also determined and is reported in Table 2.

**Table 2:** Putative gene identifications for SNP loci

Locus No.	Gene name	Gene function	SNP location and effect
1	Atrial Natriuretic Peptide	Hormone activity; regulation of blood pressure	3′ UTR
2	Chymotrypsinogen	Hydrolase activity; digestion	Coding (CGT to CGC) Arg To Arg (Synonymous)
3	Ribosomal protein S2	Protein synthesis	ACT to ACC Thr to Thr (Synonymous)
4	Translationally controlled tumor protein	Transcription factor binding; negative regulator of apoptosis	3′ UTR
5	Ribosomal protein	Protein synthesis	Coding (GAG to GGG) Glu to Gly (Nonconservative)
6	Guanine nucleotide binding protein (G‐protein), beta polypeptide 2 like 1; also called RACK1	Receptor of activated protein kinase C1 (RACK1); apoptosis	Coding (ACC to ACT) Thr to Thr (Synonymous)
7	NACA; nascent polypeptide‐associated complex subunit alphalike protein	DNA binding; cardiac development	Coding (GTA to GAA) Val to Glu (Nonconservative)
8	Titin cap	Sarcomere assembly; muscle	Coding (GAG to GAC) Glu to Asp (Nonconservative)
9	SNP name 1176_169; Probable intergenic region; No annotated gene (nearest neighbor gene gamma‐aminobutyric acid (GABA) receptor type B subunit 2)	Unknown	Intergenic
10	60S ribosomal protein L6 (RPL6)	Protein synthesis; translation	Coding (GGG to GGC) Gly to Gly (Synonymous)
11	Parvalbumin	Calcium binding; muscle	Coding (GCT to GCC) Ala to Ala (Synonymous)
12	Actin‐binding LIM protein family 3; SNP located in intron	Cytoskeleton organization; muscle	Intron
13	Myoglobin	Oxygen binding	3′ UTR
14	60S ribosomal protein L35	Protein synthesis; translation	Coding (GGC to GGT) Gly to Gly (Synonymous)
15	40S ribosomal protein S17	Protein synthesis; translation	Coding (AGT to AGC) Ser to Ser (Synonymous)
16	Cytochrome oxidase I (COXI)	Electron transport chain; Mitochondrially encoded	Coding (GGC to GGT) Gly to Gly (Synonymous)
17	Cytochrome B	Electron transport chain; Mitochondrially encoded	Coding (CTC to CTT) Leu to Leu (Synonymous)
18	SLC25A	Phosphate carrier; mitochondrial localization	Coding (GCG to GCC) Ala to Ala (Synonymous)
19	HD domain containing 2 (HDDC2); SNP located in intron	Function Unknown; probable mitochondrial localization	Intron
20	Warm acclimation‐related protein (Wap65); Hemopexin	Heme binding; oxidative stress	Coding (GCG to GTG) Ala to Val (Nonconservative)
21	Glyceraldehyde 3 phosphate dehydrogenase (GAPDH)	Glycolysis	3′ UTR
22	Tropomyosin	Muscle contraction	Coding (GAA to GAG) Glu to Glu (Synonymous)
23	Lactate dehydrogenase_B (SNP 654)	Glycolysis	Coding (TCC to GCC) Ser to Ala (Nonconservative)
24	Chymotrypsin‐C	Pancreatic peptidase	Coding (GAA to GAG) Glu to Glu (Synonymous)
25	Nucleoside diphosphate kinase 1	Biosynthesis; nucleotide synthesis	Coding (GAG to GAC) Glu to Asp (Conservative)
26	Lactate dehydrogenase‐B (SNP 1033)	Glycolysis	Coding (GCC to GAC) Ala to Asp (Nonconservative)
27	Hemoglobin alpha	Oxygen transport	Coding (CCA to GCA) Pro to Ala (Nonconservative)
28	Cytochrome p450 (Cyp3A4)	Metabolism; Monooxygenase	Coding (ATC to GTC) Ile to Val (Conservative)
29	14‐3‐3 zeta	Signaling	3′ UTR
30	Hemoglobin beta	Oxygen transport	Coding (AGT to AGC) Ser to Ser (Synonymous)
31	Nucleoside diphosphate kinase 2	Biosynthesis; nucleotide synthesis	3′ UTR
32	Intergenic 1173	Unknown	Noncoding

### Genetic analysis

Genepop 4.0.10 (Raymond and Rousset 1995) was used to calculate *F*_IS_ values and conduct an exact test for Hardy–Weinberg equilibrium (HWE) using the complete enumeration method, and test for heterozygote excess and deficiency for each SNP locus in each location. In addition, this program was used to test for cytonuclear disequilibrium among the SNPs. Genepop computes disequilibrium using a log‐likelihood ratio test (G‐statistic). Markov chain parameters were set to the defaults (dememorization number = 1000; 100 batches; 1000 iterations per batch). The Black and Krafsur procedure, as implemented in Genetix 4.05.2, was used to test each pair of SNP loci among individuals from each population for evidence of linkage disequilibrium (Cockerham and Weir 1977; Belkhir et al.^1999^). We compared these parameters among populations to ask the question whether they differed between populations located outside and within the putative contact zone that we previously defined using microsatellite markers (McKenzie et al. 2015).

### Clinal analysis

ClineFit v0.2 (Porter et al. 1997) was used to estimate the width, center, and minimum and maximum frequencies of asymptotic polymorphisms (*p*_min_ and *p*_max_, respectively) of all 32 SNPs. For these analyses, the location of our most southern sampling location (Location 15: Sapelo Island, Georgia) was set to zero and the distance to each sampling location was measured using ArcGIS ArcMap 10 (ESRI 2010). Clines were fit using new cline shape parameters (as opposed to testing the fit of the data to a predefined set of parameters), and each run was initialized with rough settings (as opposed to the user providing starting values for the center, width, and asymptotes of each SNP). ClineFit also calculates two‐unit support limits, which are analogous to 95% confidence intervals, for each parameter. Clines were plotted by fitting a sigmoid curve on the presented data using the slope (=4/width; Endler 1977) and center as estimated with ClineFit, as well as the height (maximum allele frequency − minimum allele frequency) as calculated from the data itself.

The resulting cline width (*w*) estimations were then used to calculate the number of generations that have elapsed under the assumption of a neutral cline using the equation:

(1) $$T={\left(\frac{w}{\left(\sqrt{2\pi }\right)\sigma }\right)}^{2}$$

where *T* represents the number of generations since contact under the assumption of a neutral cline, and *σ* represents dispersal per generation (Barton and Gale 1993).

We also used the SNP data to calculate an estimate of dispersal as described by Barton and Gale (1993). First, we estimated average pairwise linkage disequilibrium ($\overset{¯}{D}$) using:

(2) $$\;\text{var}\;\left(z\right)=\frac{1}{2n}\left(\overset{¯}{z}(1-\overset{¯}{z})-\;\text{var}\;(p)\right)+\frac{1}{2}\left(1-\frac{1}{n}\right)\overset{¯}{D}$$

where *z* = hybrid index as calculated using the program *introgress* (Gompert and Buerkle 2009, 2010) for individuals from the marsh located at the center of the mitochondrial cline (Location 9: Metedeconk, NJ) using individuals from Location 1 (Wiscasset, ME) and Location 15 (Sapelo Island, GA) as northern and southern parental types, respectively. Thus, $\overset{¯}{z}$ and var (*z*) are the mean and variance of the hybrid index for these individuals, respectively, and *n* = the number of loci. The variance of allele frequency across the n loci was calculated as $ar\left(p\right)=1/n\sum {\left(pi-\overset{¯}{p}\right)}^{2}$. The resulting value for $\overset{¯}{D}$ was then substituted into:

(3) $$\overset{¯}{D}=\frac{\sigma^{2}}{{r\overset{¯}{w}}^{2}}$$

where *r* = recombination rate (0.5) and $\overset{¯}{w}$ = mean cline width. Equation (3) was subsequently solved for *σ*.

Cfit6 (Gay et al. 2008) was then used to test clines for coincidence and concordance by first fitting a model in which all clines were constrained to a common center and width and then comparing the likelihood of this model to one in which all clines parameters were allowed to vary independently.

### Hybrid index analysis

The program *structure* 2.3.4 (Pritchard et al. 2000; Falush et al. 2007) was used to analyze multilocus SNP genotypes to identify pure parental and admixed individuals and to ultimately define the borders of the hybrid zone. The program was run set to the defaults as per the recommendation of the authors. A 50,000 iteration burn‐in was followed by 150,000 Markov chain Monte Carlo iterations for parameter estimation. The number of clusters (*k*) tested ranged from one to ten, and ten replicate analyses for each value of *k* were performed. The most likely number of clusters was then determined using Evanno et al.'s (2005) statistic *Δk* as calculated by *structure harvester* (Earl and vonHoldt 2012). Pure northern individuals were then identified as having a *q* ≥ 0.90, pure southern as those having *q* ≤ 0.10, with all remaining individuals being characterized as admixed (0.10 < *q* < 0.90; Vaha and Primmer 2006).

The program *introgress* was used to calculate maximum‐likelihood estimates of hybrid index (Buerkle 2005; Gompert and Buerkle 2009). Briefly, this program uses user‐defined parental populations to calculate the hybrid index of admixed individuals. For this analysis, we used the output of the *structure* analysis (above) with the identified most likely number of clusters (*k *=* *2, see Results section below) to choose the populations to define as parental, with the goal of selecting populations that were geographically proximate to the contact zone but that contained a low frequency of admixed individuals. As a result of these analyses, Location 5 (Cheesequake, NJ) was defined the most likely northern parental population and Location 12 (RUMFS, NJ) was defined the most likely southern parental population. Location 9 (Metedeconk, NJ) was identified as the center of the zone of admixture (Fig. 1; Table 1). The resulting hybrid index values were then combined in a histogram in 0.10 increment bins. Hartigan's dip test (Hartigan 1985; Hartigan and Hartigan 1985), as executed in the R statistical computing environment (R Development Core Team 2009), was then used to determine the general shape of the frequency distribution of hybrid indices (unimodal vs. bimodal).

## Results

### Linkage disequilibrium, heterozygote deficit, and cytonuclear disequilibrium

There was little evidence of significant linkage disequilibrium (LD) among loci (Table S2), with only the two most northern populations (Locations 1 and 2), demonstrating any evidence of pairwise linkage disequilibrium among loci following correction for false discovery rate (FDR; Pike 2010). All of these instances had positive values of Rij, suggesting that alleles from like parental types are more often found together. Note that our analysis contains data from two SNPs within the *Ldh‐B* gene that are located less than 3 kb apart in the genome. We would expect this physical linkage to produce linkage disequilibrium between these SNPs in most populations. However, this pattern was only detected in the two most northern populations (Table S2). This finding is consistent with previous work on this gene in *F. heteroclitus*, which has detected significant recombination within the *Ldh‐B* gene (Bernardi et al. 1993; Schulte et al. 1997). The only other SNPs that are located on a single scaffold in the killifish genome are SNPs in two hemoglobin genes. These genes are located approximately 40 kb apart, and no FDR‐significant linkage disequilibrium was detected between them in any population. Because FDR correction can be conservative, we also examined the uncorrected data and obtained similar patterns (Tables S3 and S4). The two *Ldh‐B* SNPs were in linkage disequilibrium in all but the three southernmost populations, and the two hemoglobin genes were in significant linkage disequilibrium in 9 of the 15 populations studied. Significantly, more loci had positive Rij across all populations (suggesting that alleles from like parental types are more often together) than negative Rij (Mann–Whitney *U*‐test *P* < 0.0001). However, there was no evidence of elevated LD within the putative contact zone, as there was no significant difference in the number of loci exhibiting positive Rij in hybrid zone populations compared to locations to the north or south of the zone (Kruskal–Wallis nonparametric ANOVA *P* = 0.3473).

**Figure 2:**
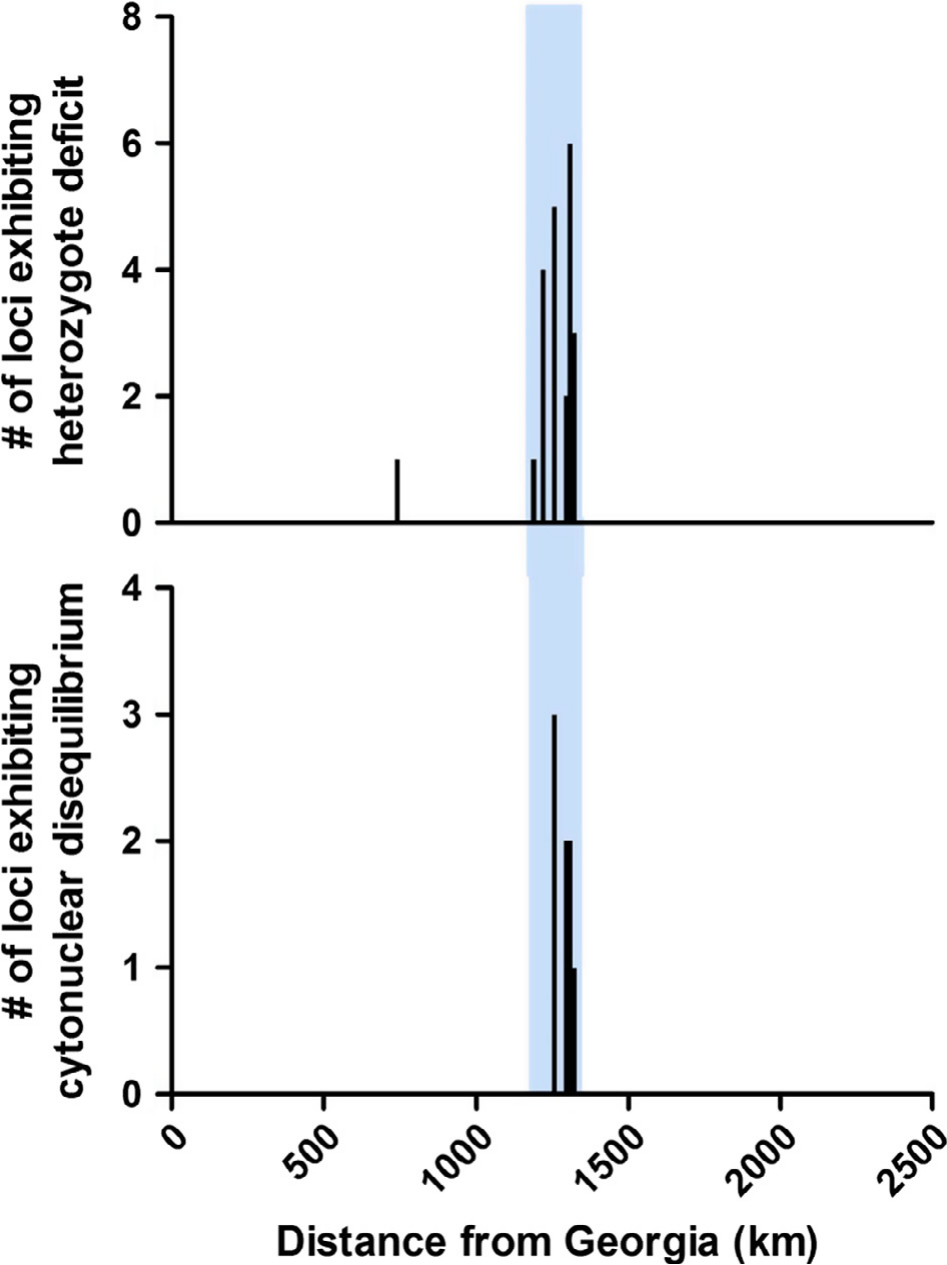
Number of loci exhibiting significant heterozygote excess (A) or cytonuclear disequilibrium (B) in each population. Blue bar indicates the location of the putative contact zone as defined in McKenzie et al. (2015) and includes locations 5–12 in Table 1 (all locations in New Jersey).

Of the 30 nuclear SNPs genotyped, ten showed significant deviations from HWE following adjustment for false discovery rate for at least one of the 15 locations sampled, with a total of 24 significant deviations following FDR adjustment for all locus and location combinations. Of these 24 significant deviations, 23 were heterozygote deficits and all occurred among individuals from collection locations within the putative contact zone (Fig. 2A). Similar patterns were evident in the uncorrected data, in which 20 loci deviated significantly from HWE in at least one of the 15 locations sampled. These deviations were dominated by heterozygote deficits (92% had positive values of *F*IS), with the majority of the deviations (78%) occurring in populations within the borders of the contact zone (Table S5).

There was also evidence of elevated cytonuclear disequilibrium within the putative contact zone (Fig. 2B). Populations from marshes peripheral to the contact zone showed no evidence of loci in disequilibrium with the two mitochondrial SNPs genotypes, while four marsh populations from northern New Jersey (at the center of the putative contact zone as well as the center of the mitochondrial cline) exhibited significant associations between mitochondrial and nuclear genotypes. However, the specific loci involved differed among marshes. In location 5 (Cheesequake, NJ), the SNP in NACA was found to be significantly associated with the SNP in mitochondrial cytochrome B. In locations 6 (Belford Creek, NJ) and 7 (Sandy Hook, NJ), the SNP in titin cap was in significant disequilibrium with both of the mitochondrial SNPs (cytochrome B and cytochrome oxidase subunit I). Lastly, in location 9 (Metedeconk, NJ) the nuclear SNP in G‐protein beta polypeptide (RACK1) was in disequilibrium with mitochondrial COXI, and a phosphate carrier protein (SLC25A3), a nuclear‐encoded mitochondrial gene, was in cytonuclear disequilibrium with both mitochondrial SNPs.

### Cline parameters

**Figure 3:**
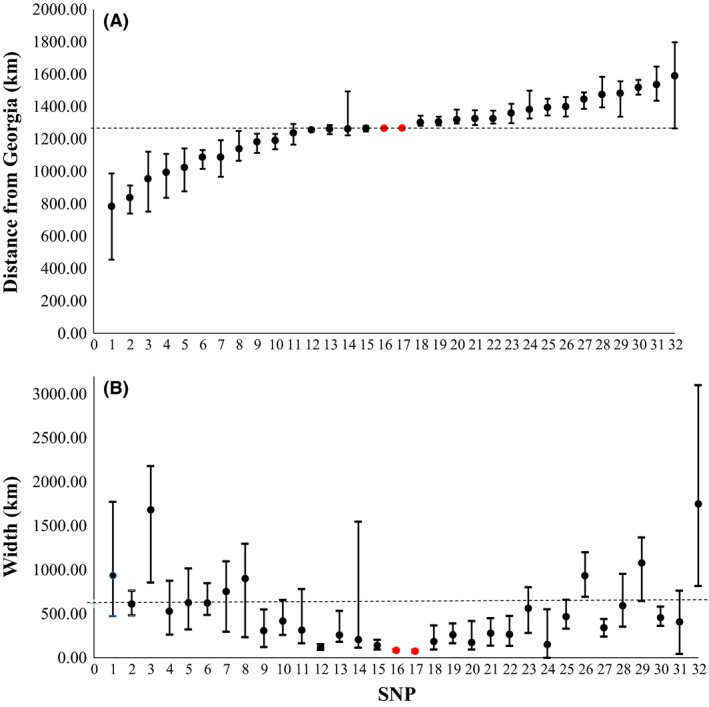
Cline centers (A) and widths (B). Mitochondrial loci highlighted in red. Loci are arranged in the order of increasing distance of the cline center from Georgia. Numbering on the *x*‐axis corresponds to SNP locus numbers as designated in Table 2. Error bars represent the 2‐unit support limits of the estimates. (A) Dashed line illustrates center of mitochondrial clines. (B) Dashed line represents width of neutral cline calculated from mark–recapture estimate of dispersal. Clines with an upper 2‐unit support limit below the dashed line are putatively non‐neutral.

Calculated cline centers (arranged in the order of increasing distance from Georgia) are shown in Figure 3A, and the corresponding cline widths in Figure 3B. See Figure S1 and Table S6 for cline shapes and parameters. It is possible to predict the expected width of a neutral cline resulting from secondary contact making some assumptions about the time since contact and dispersal distance per generation. *Fundulus heteroclitus* has a generation time of approximately 1 year, as individuals are capable of breeding at 1 year of age, and continue breeding each year thereafter up to their typical maximum lifespan in nature of approximately 2–3 years (Abraham 1985). We assumed that the commencement of the last glacial retreat marks the beginning of contact between the two subspecies, and thus, it has been approximately 15,000 years, or 15,000 generations, since contact occurred. Within subtidal creeks, mark–recapture data suggest that individual *F. heteroclitus* can move as much as 2 km within a season in a tidal creek (Fritz et al. 1975), and as much as 3.7% of a population may travel greater than 1 km within a complex marsh system consisting of marsh pools and intertidal and subtidal creeks within a single year (Able et al. 2012). Given the large population sizes of *F. heteroclitus* in these marshes (on the order of 10^5^ individuals; Adams et al. 2006), this is consistent with broad‐scale movements of substantial numbers of fish within large marshes. However, little is known about dispersal between marshes, which would require dispersal across potentially unfavorable habitat along the coast. We used 2 km per generation as a conservative estimate of dispersal distance (*σ*) because we found no evidence of genetic isolation by distance between marshes along the coast at this scale (data not shown). Our findings were similar to those of Brown and Chapman (1991), suggesting that dispersal distances along the coast must be at least of this magnitude. Using these assumptions, we can calculate the number of generations since secondary contact, assuming all clines are neutral. The calculated number of generations since secondary contact ranges from 225 for the steepest cline (in the mitochondrial genome) to 121,749 generations for the shallowest cline (in locus 1173) (see Table S6 for values for all clines). Alternatively, rearrangement of equation (1) allows us to predict a cline width (*w*) of 614 km if diffusion is neutral, assuming that secondary contact occurred ~15,000 years ago, following the last glacial retreat (indicated with a dashed line on Fig. 3B). The widths of the clines produced by 14 of the SNP loci are significantly less than this predicted value (SNPs 9, 12, 13, 15, 16, 17, 18, 19, 20, 21, 22, 24, 27, 30), while four loci (SNPs 3, 26, 29, 32) have clines that are significantly wider than would be predicted under a neutral model (Fig. 3B; Table S6).

As an alternative approach to estimating dispersal distance, we utilized tension zone theory to calculate a genetic estimate of dispersal distance using average pairwise linkage disequilibrium (see equations (2) and (3)). This approach yielded a predicted per generation dispersal distance of 14.5 km emphasizing the conservative nature of the 2 km per generation dispersal distance used above. Thus, our conclusions relating to the expected neutral cline width and ultimately levels of selection on the narrow clines are, if anything, similarly conservative underestimates.

### Cline coincidence and concordance

We used an Akaike information criterion **(**AIC) approach to assess cline coincidence and concordance. A model in which all clines were constrained to have the same center and width did not fit the data as well as a model in which the cline centers and widths were allowed to vary independently (lnLikelihood = −7669.49, *k* = 2, AIC = 15342.98 vs. lnLikelihood = −7041.96, *k* = 28, AIC = 14139.92, respectively), a pattern which is obvious in Figure 3A and B. However, a subset of loci had cline widths that did not meet neutral expectations (Fig. 3B) and also had two‐unit support limits of their centers within 10 km of the two‐unit support limits of the mitochondrial cline centers (Table S6). This subset of SNPs (in the actin‐binding LIM protein, myoglobin, 40S ribosomal protein S17, SLC25A3, and HDDC2 genes [SNPs 12, 13, 15, 18, and 19, respectively]) thus have clines that are coincident and concordant with the mtDNA clines.

### Structure analysis

**Figure 4:**
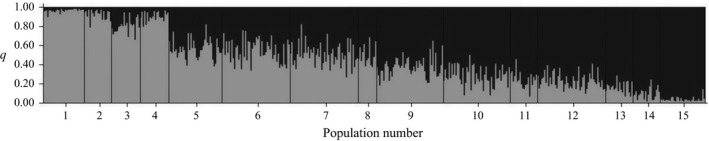
Admixture proportions (*q*) as calculated with *structure*,* k* = 2. Grey denotes the proportion of an individual's multilocus genotype inherited from the northern type (*Fundulus heteroclitus heteroclitus*); Black bars indicate the proportion contributed from the southern type (*Fundulus heteroclitus macrolepidotus*). Population numbers correspond to those in Table 1.

The *structure* analysis of the 15 populations sampled spanning the entire range of the distribution of *F. heteroclitus* confirmed the presence of two hybridizing groups, with the *Δk* for *k* = 2 far exceeding those reported for the alternate numbers of clusters tested. 11.57% of the individuals sampled had admixture proportions >0.9 and the majority of these originated from the northerly located populations (Table 1, locations 1–4; Fig. 4). A total of 10.12% of the individuals sampled had admixture proportions <0.1 and were derived from mainly the southern populations (locations 13–15). The remaining individuals (78.31%) had intermediate admixture levels and were collected primarily between the locations 12 and 5 (RUMFS and Cheesequake, NJ, respectively).

### Hybrid index

**Figure 5:**
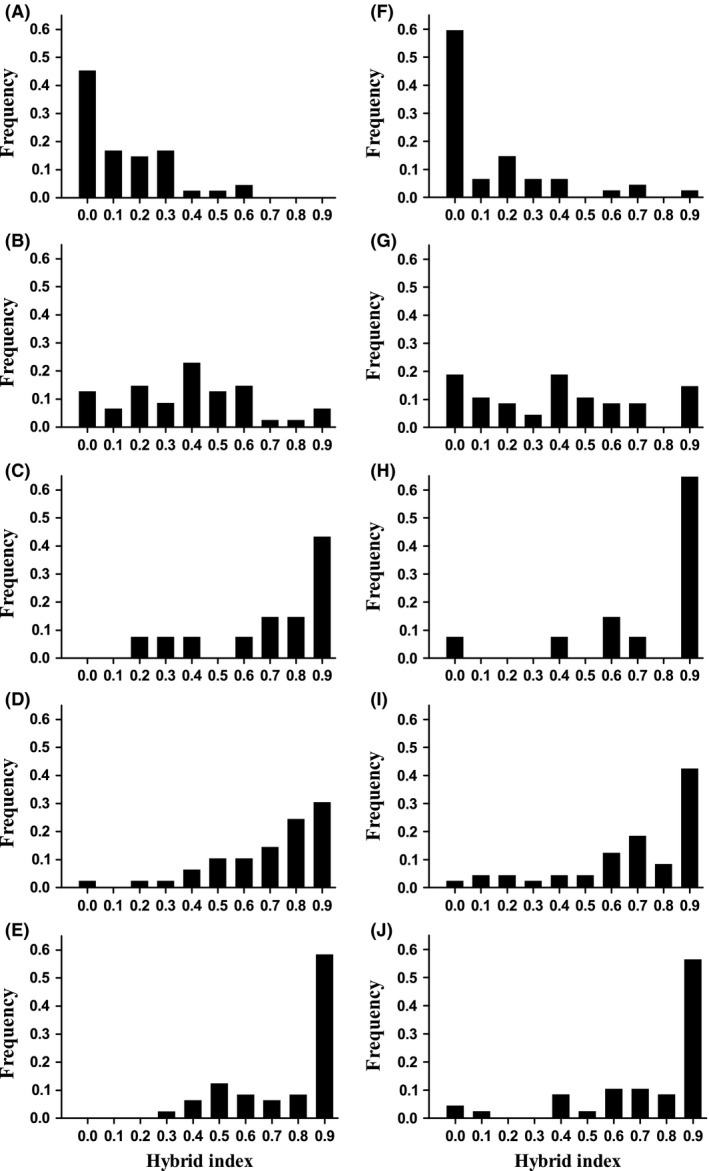
Histogram of hybrid index values of individuals collected from hybrid zone populations calculated using *introgress*, with individuals collected from Cheesequake and RUMFS designated as northern and southern parental types, respectively. Hybrid index values were calculated using (A–E) all 30 nuclear SNPs and also calculated using (F–J) a subset of five SNPs. A hybrid index value of 0 indicates a pure southern individual; a hybrid index value of 1 indicates a pure northern individual. Populations are arranged from south to north. Panels A&F, location 11; Panels B&G, location 10; Panels C&H, location 9; Panels D&I, location 7; Panels E&J, location 6. Note that hybrid index was not calculated for location 8 because of a limited sample size.

**Figure 6:**
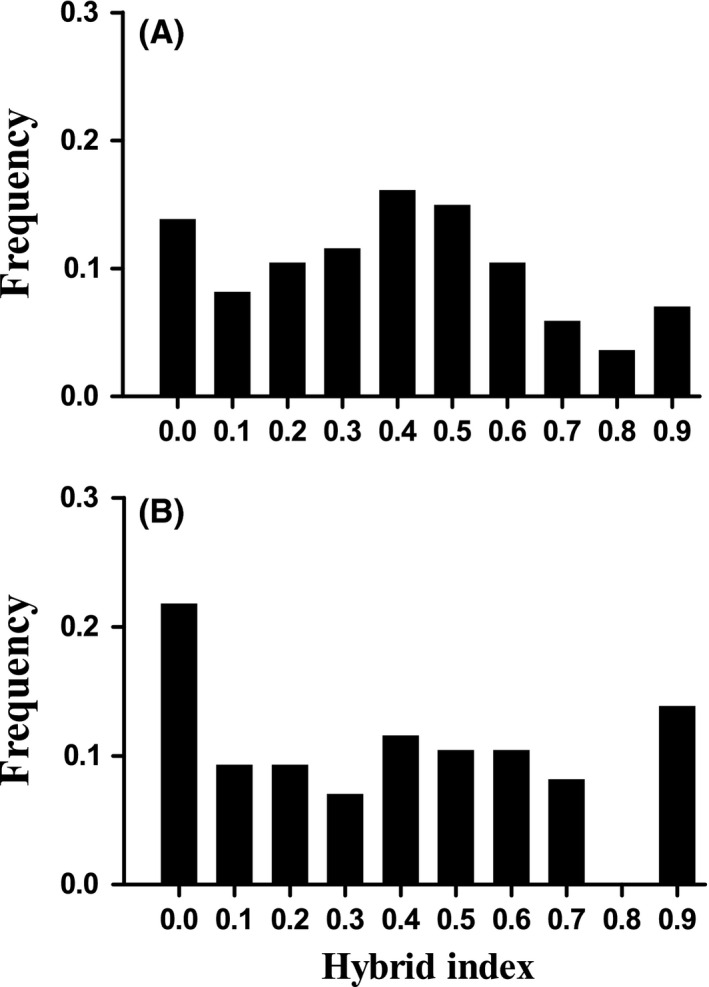
Frequency distribution of hybrid indices as calculated for individuals sampled from Metedeconk across two seasons. Hybrid index values were calculated using (A) all 30 nuclear SNPs and using (B) a subset of five SNPs (locus numbers 12, 13, 15, 18, and 19, Table 2). A hybrid index value of 1 indicates a pure northern individual while a hybrid index value of 0 indicates a pure southern individual.

The results of the *structure* analysis (Fig. 4) clearly indicated a transition from a pure northern genotype to an admixed genetic background at location 5 (Cheesequake). In the south, there is a more gradual transition from the zone of admixture, with locations 11, 12, and 13 exhibiting average admixture proportions (*q*) greater than 0.1, indicating the presence of some admixed individuals. As individuals from more proximate populations are more likely to resemble individuals that are making a genetic contribution to individuals in the hybrid zone than are individuals from the extremes of the species distribution, we chose to use populations from the identified edges of the zone of secondary contact in the training dataset for the subsequent calculation of hybrid index using the program *introgress*. Thus, we used location 5 (Cheesequake, NJ) as the putative northern parental population (mean *q* = 0.569 compared to mean *q* = 0.906 for location 4). At southern end of the secondary contact zone, locations 11, 12 and 13 had mean *q* of 0.247, 0.236, and 0.167, respectively. We selected location 12 (RUMFS, NJ) as the putative southern parental population because it is geographically proximate to the secondary contact zone and because we had the highest sample size for this location, allowing a more accurate estimation of parental allele frequencies. However, note that similar results are obtained when any of these populations are used as the southern parent. We calculated hybrid index using both the complete set of nuclear SNPs and the subset of five SNPs that had cline shapes that were coincident and concordant with the mtDNA clines (actin‐binding LIM family protein 3, myoglobin, 40S ribosomal protein S17, SLC25A3, and HDDC2; loci 12, 13, 15, 18, 19; Fig. 3; Table S6) to determine whether these SNPs showed different patterns in hybrid index compared to the complete data set. Figure 5 shows the frequency distribution of the hybrid indices in the admixed populations for both the complete set of SNPs (Fig. 5A–E) and the subset of five SNPs (Fig. 5F–J), arranged from south to north. As was the case with the *structure* analysis, there was a shift from a more southern hybrid index (e.g., Fig. 5A and F) to a more northern hybrid index with increasing latitude (Fig. 5C–E and H–J). In location 9 (Metedeconk, NJ), the pattern of the distribution of hybrid indices was flat or unimodal when all SNPs were used in the hybrid index calculation (Fig. 5B) and the deviation from unimodality is even clearer when the subset of markers were used (Fig. 5G). To increase our power to determine the shape of this frequency distribution, we combined these data with additional multilocus genotype information from 39 fish sampled the subsequent Fall (2009). The two samples (Summer 2008, Fall 2009) were not significantly different (*F*_ST_ = 0.00051; *P* > 0.05), justifying the merging of the two groups. The combined data did not deviate from unimodality for hybrid indices calculated including all 30 nuclear SNPs (Fig. 6A; *D* = 0.0364; *P* = 0.5589). However, for hybrid indices calculated using the subset of five nuclear SNPs, the combined data revealed a trend toward bimodality (Fig. 6B). Notably, the two extreme bins contained 21.59% and 13.64% (more than a third) of the observations. However, Hartigan's dip test did not detect a significant departure from unimodality (*D* = 0.0463; *P* = 0.1774).

## Discussion

By examining genetic variation in 30 nuclear and 2 mitochondrial SNPs in *F. heteroclitus*, we have demonstrated that: (1) populations from marshes located in the center of the putative contact zone between *F. heteroclitus* subspecies (i.e., along the coast of New Jersey) exhibit higher levels of heterozygote deficit than do populations from marshes outside of the zone, (2) there is some evidence of cytonuclear disequilibrium within the contact zone, (3) there is a subset of very narrow clines in nuclear SNPs that are coincident and concordant with the mtDNA cline, and (4) multilocus analysis using this subset of markers suggests a non‐unimodal pattern of genotypic frequencies for these loci in the population at the center of the mtDNA cline, suggesting that contemporary forces such as exogenous selection, endogenous selection, and/or assortative mating may be operating in this hybrid zone. This latter observation is consistent with our previous findings of a bimodal distribution of hybrid indices calculated from microsatellite markers (McKenzie et al. 2015).

### Cline steepness and position

The widths of 14 of the 32 clines were significantly less than that predicted by a model of neutral diffusion, using a conservative estimate of dispersal based on mark–recapture data. These results are broadly consistent with the results of Strand et al. (2012) who found a small subset of loci with extremely steep clines among the 310 loci that they surveyed in *F. heteroclitus*. We detected a much higher proportion of steep, putatively selected clines because we targeted loci with high levels of differentiation between the geographically extreme populations, which biases our sample toward loci with steeper clines, but allows us to direct our focus to loci that are more likely to be experiencing selection.

The widths of these putatively non‐neutral clines ranged from 75 km (cytochrome b) to 457 km (hemoglobin beta). Clines this steep would have to have been formed between approximately 225 and 1300 years ago in the absence of selection, assuming a dispersal distance (*σ*) of 2 km per generation. However, because of the assumptions inherent in clinal analyses, it is extremely challenging to determine whether these unusually steep clines are, in fact, being maintained by selection. In addition, the possibility that the observed clines are the result of neutral processes such as allele surfing as populations expanded into previously glaciated regions must also be considered (Excoffier and Ray 2008). Information about the functional roles of the loci exhibiting steep clines has the potential to help generate hypotheses regarding the forces maintaining these clines. Clines in functionally related loci that are coincident, concordant, and steep are of particular interest because they are more likely to be responding to similar forces.

Although functional associations among the genes containing putatively non‐neutral SNPs could support the hypothesis that exogenous and/or endogenous selection may be shaping these clines, it is crucial to note that the SNPs we have interrogated may simply be markers of genomic locations, rather than functionally important sites, per se. Indeed, many of these SNPs are in 3′ untranslated regions or are synonymous substitutions that do not change the amino acid sequence (Table 2). However, functional information regarding the genes containing these marker SNPs is still likely to be informative, because *F. heteroclitus* linkage groups appear to be small (Bernardi et al. 1993; Schulte et al. 1997), suggesting that the marker SNPs may be linked to functionally important SNPs within the same gene. This conclusion is supported by our failure to detect significant linkage disequilibrium between the hemoglobin alpha and beta genes, which are located within 40 kb on the same genomic scaffold.

As has been previously documented (Strand et al. 2012), the steepest of the *F. heteroclitus* clines are in mtDNA. Several of the nuclear SNPs with steep clines coincident and concordant with the mtDNA clines are located in genes involved in oxidative metabolic processes (Table 2; Table S6), including the only two nuclear‐encoded mitochondrial proteins in our SNP panel: SLC25A3 and HDDC2. Although the function of HDDC2 is poorly understood, the function of SLC25A3 is well characterized. It is responsible for the transport of phosphate groups from the cytosol into the mitochondrial matrix (Becker et al. 2003), which is essential for the function of the mitochondrial F_1_F_0_ATPase. Another of these coincident and concordant SNPs is within the myoglobin gene. Myoglobin is an oxygen storage molecule, and is thus involved in the transport of oxygen from the environment to the mitochondria in muscle, and therefore is critically involved in oxidative metabolism. Interestingly, the allozymes of malate dehydrogenase (MDH), a locus which was not examined here, also exhibit an extremely steep cline that is coincident and concordant with the mtDNA cline in *F. heteroclitus* (Strand et al. 2012). This isoform of MDH is localized in the mitochondrion, where it participates in the citric acid cycle which feeds electron carriers to the electron transport chain of the mitochondria. The functional association between these nuclear‐encoded SNPs and mitochondrial processes coupled with the coincident and concordant clines observed between the mitochondrial and nuclear genomes for these loci is suggestive of the action of some form of endogenous or exogenous selection on mitochondrial processes in *F. heteroclitus*.

Several of the other genes with putatively non‐neutral clines are also involved in functions associated with oxidative metabolism. For example, glyceraldehyde‐3‐phosphate dehydrogenase (GAPDH) is a glycolytic enzyme that is critical for supplying pyruvate to the mitochondrion. Variation in the expression of glyceraldehyde‐3‐phosphate dehydrogenase is associated with variation in cardiac metabolism between *F. heteroclitus* subspecies (Podrabsky et al. 2000) and has been suggested to be under selection in this genus (Pierce and Crawford 1997).

Similarly, there were steep, non‐neutral clines in SNPs in both hemoglobin alpha and beta. Hemoglobin is also involved in oxidative metabolism, as it is responsible for oxygen transport in the blood. Differences in hemoglobin–oxygen affinity are associated with differences in swimming performance between *F. heteroclitus* subspecies (DiMichele and Powers 1982b; Fangue et al. 2008), which is a good fitness proxy in fishes (e.g., Dalziel et al. 2012). In addition, there was a steep, non‐neutral cline in a SNP in a gene called warm acclimation‐related protein, which is known to function in the response to oxidative stress (Kikuchi et al. 1995; Sarropoulou et al. 2010).

Taken together, these data suggest that oxidative processes may be responding to endogenous and/or exogenous selection in *F. heteroclitus*. However, five of the SNPs with non‐neutral clines do not have functions that are obviously associated with oxidative metabolism. Actin‐binding LIM family protein 3 and tropomyosin have functions related to muscle contraction and are plausibly associated with swimming performance. 40S ribosomal protein L17 is likely involved in protein translation, and chymotrypsin‐C is involved in extracellular calcium regulation. SNP 1176 is located in an intergenic region very close to a gene coding for the gamma‐aminobutyric acid receptor (type B; GABA_B_ receptor). The function of the GABA_B_ receptor has received limited study in fishes, but it is expressed in the central nervous system and the available data suggest that it is involved in the control of locomotion, among other functions (Tegner and Grillner 2000).

Interestingly, there are a number of loci that demonstrate deviations from neutral expectations across multiple analyses. There are five loci with significant deviations from HWE that have high positive Fis values at 4 or more sites: 9, 12, 19, 20, and 29 (Table S5). Of these, three have narrow cline widths (9, 12, and 20, Table S6), and the cline in locus 12 is coincident and concordant with the cline in mitochondrial DNA. As discussed above, these loci (the GABA_B_ receptor, actin‐binding LIM protein, and warm acclimation‐related protein, respectively) all have functions associated with exercise and oxidative stress. In addition, locus 19, the mitochondrial phosphate transporter, has a narrow cline width and a cline center concordant with the mitochondrial DNA cline, and also exhibits cytonuclear disequilibrium, again indicating oxidative metabolism as a potential target of selection across this contact zone. The observation that no single locus exhibits cytonuclear disequilibrium across multiple populations within the contact zone also suggests that a process such as allele surfing is unlikely to be the cause of the clinal patterns observed in this species.

Two of the SNPs examined in this study are in the gene coding for lactate dehydrogenase‐B (*Ldh‐B*), which has been the subject of intensive investigation in this species (Powers et al. 1979; DiMichele and Powers 1982a,b; Place and Powers 1984; Paynter et al. 1991; DiMichele and Westerman 1997). Functional studies suggest that the alternative northern and southern alleles at this locus differ in kinetic properties (Place and Powers 1984) and may influence whole‐organism performance (Powers et al. 1979; Fangue et al. 2008). However, the cline width at this locus did not differ from the neutral expectation, exhibiting a fairly gradual change with latitude and a cline center slightly north of the mitochondrial cline. We also compared the shape of the contemporary cline generated from our SNP data to a cline generated using the LDH‐B allozyme frequency information presented by Powers & Place (1978). One of the SNPs examined here is responsible for the two allozymes at this locus (Powers & Place 1978). Comparison of the contemporary and historical clines indicates that the LDH‐B cline center has remained the same over the last three and a half decades (Fig. S2). These results are consistent with another recent analysis of this cline (Bell et al. 2014), which also found that this cline has not shifted in position despite strong evidence of temperature increases over time (Bell et al. 2014).

### Evidence of selection against hybrids

A greater proportion of loci exhibited significant departures from Hardy–Weinberg equilibrium due to elevated levels of heterozygote deficit in marshes in the contact zone compared to those outside of the contact zone. Although departures from Hardy–Weinberg equilibrium can be caused by a variety of factors including population subdivision, natural selection, avoidance of heterospecific mating, or assortative mating, many of these processes would be expected to have effects on a whole‐genome scale. In *F. heteroclitus*, however, many loci that differed between the subspecies were in Hardy–Weinberg equilibrium in populations in the contact zone, clearly indicating that the observed heterozygote deficits are the result of locus‐specific phenomena, rather than genomewide forces. Only selection (endogenous, exogenous, or a combination) or assortative mating based on a phenotype specified by a small number of loci would be predicted to have locus‐specific effects such as those observed here.

One of the SNPs demonstrating significant heterozygote deficit was in the 3′ untranslated region of the signaling protein 14‐3‐3 zeta, which is known to be important in osmosensing signal transduction in the gill (Kültz et al. 2001). This is significant because the northern and southern subspecies of *F. heteroclitus* differ in salinity tolerance, particularly during development (Scott et al. 2004; Whitehead et al. 2011), and there are steep replicated clines in mtDNA and microsatellites in *F. heteroclitus* across salinity gradients in several large estuaries along the Atlantic coast, which is consistent with the action of environmental selection for freshwater tolerance in this species (Whitehead et al. 2011). Given this variation, it is possible that adult *F. heteroclitus* might prefer habitats of differing salinity for laying their eggs, which opens the possibility that assortative mating might be influencing genetic patterns at this locus. However, this locus did not exhibit a particularly narrow cline, suggesting that it is not being influenced by forces similar to those affecting the mtDNA clines.

Three of the SNPs with strong heterozygote deficits (atrial natriuretic peptide, warm acclimation‐related protein, HDDC2) have functions that are likely associated with oxidative metabolism. As previously mentioned, HDDC2 is a nuclear‐encoded but mitochondrially localized protein of unknown function. Warm acclimation‐related protein is a hemopexin involved in coping with oxidative stress that is known to increase in expression in response to a variety of stressors (including temperature) in fish (Pierre et al. 2010). Atrial natriuretic peptide is a hormone involved in osmoregulation that also has effects on aerobic metabolism in skeletal muscle (Engeli et al. 2012). These data suggest that patterns of genetic variation at these three loci might, at least in part, be shaped by endogenous selection against hybrids due to similar forces to those acting on the mtDNA clines.

The final line of evidence consistent with moderate reproductive isolation acting within this contact zone is the relatively flat or bimodal distribution of genotypic classes within the population at the center of the mtDNA cline. This pattern was clearest for a subset of five loci found to be coincident and concordant with the mitochondrial clines (actin‐binding LIM protein, myoglobin, 40S ribosomal protein S17, SLC25A3, and HDDC2), when two collection seasons are combined. Inspection of Fig. 6B suggests a reduced frequency of advanced‐generation backcrosses, and a slight excess of pure parental types in a centrally located marsh. At this time, and using this subset of markers, we do not have enough evidence to conclusively state that the distribution is non‐unimodal. However, the Hartigan's dip test is known to be extremely conservative at smaller sample sizes, and inspection of the frequency distribution of hybrid indices suggests a flat or bimodal pattern, which could indicate that there is a moderate degree of reproductive isolation acting through locus‐specific processes in this population. Alternatively, the slight excess of pure parental types could be the result of continued immigration into the contact zone.

### Evidence for cytonuclear epistasis

Although some of the subunits of the complexes of the mitochondrially housed electron transport chain are encoded by the mitochondrial genome, the majority are transcribed from the nuclear genome and subsequently transported to the mitochondrion to form the multi‐subunit complexes of the electron transport chain. Thus, there is a functional interaction between nuclear and mitochondrially encoded genes (epistasis; Wolf et al. 2000). Such epistatic interactions are expected to promote selection favoring the occurrence of matching and thus more functionally compatible cytonuclear types within an individual. Cytonuclear incompatibilities have been implicated in hybrid breakdown in a variety of species (Liepins and Hennen 1977; Edmands and Burton 1999; Burton et al. 2006; Ellison and Burton 2008; Niehuis et al. 2008).

*Fundulus heteroclitus* subspecies differ in mitochondrial properties, particularly when acclimated to cold temperatures (Fangue et al. 2009; Dhillon and Schulte 2011; Baris et al. 2016), suggesting the possibility of functional mismatches in hybrid individuals. Four SNP‐containing loci (RACK1, NACA, titin cap, and SLC25A3) showed significant cytonuclear disequilibrium with either one or both of the mitochondrial SNPs in at least one marsh within the contact zone. However, the signal of cytonuclear disequilibrium was not particularly clear or unequivocal. Strong cytonuclear disequilibrium would be expected to produce a detectable statistical signal within populations and result in clines that are coincident and concordant with the mitochondrial cline. This is not the case for RACK1, NACA, or titin cap, but the nuclear‐encoded mitochondrial protein SLC25A3 demonstrated significant cytonuclear disequilibrium in hybrid populations and a cline center and width similar to those of the mitochondrial SNPs. This observation is consistent with the known role of SLC25A3 in mitochondrial oxidative metabolism, which is a process that requires interaction between nuclear‐encoded and mitochondrially encoded proteins.

## Conclusions

Although it can be challenging to detect the action of non‐neutral processes in situations where there are strong demographic signals of secondary contact or population expansion, particularly using sequence data alone, our data are consistent with the action of non‐neutral forces in shaping the patterns of genetic variation at a small subset of loci in *F. heteroclitus*. Detection of elevated heterozygote deficit and cytonuclear disequilibrium in populations near the center of the contact zone and the presence of a non‐unimodal pattern in multilocus genotypic frequencies in the population at the mtDNA cline center suggests that endogenous or exogenous selection against advanced‐generation backcrosses, or some form of assortative mating due to a phenotype specified by a small number of loci could be acting in this hybrid zone. The observation that several of the clines in nuclear SNPs that are non‐neutral or are coincident and concordant with clines in mitochondrial loci are in genes with functions associated with oxidative metabolism suggests that endogenous selection due to epistatic interactions or exogenous selection on processes dependent on oxidative metabolism may be a critical factor stabilizing some of the clines in this species.

## Conflict of Interest

None declared.

## Data Accessibility

Multilocus SNP genotypes have been provided for each individual from every sampling location in Table S7. All raw data will also be archived in Dryad.

## Supporting information


**Figure S1.** Clines in allele frequency for all SNP loci included in this study.
**Figure S2.** Cline in *Fundulus heteroclitus Ldh‐B* allele frequency from the current data set (shown in red) and data from Powers and Place (1978) shown in blue.

**Table S1.** Sequence and SNP location information for loci used in this study.

**Table S2.** Summary of locus pairs in significant linkage disequilibrium after FDR‐adjustment at each location.
**Table S3.** Number of loci showing deviations from HWE, LD or cytonuclear disequilibrium prior to FDR correction.
**Table S4.** Summary of locus pairs in significant linkage disequilibrium at each location.
**Table S5.** *F*_IS_ values for loci that deviated significantly from HWE.
**Table S6.** Four cline shape parameters as predicted by ClineFit, arranged by order of increasing centre (as in Fig 3).

**Table S7.** Multilocus mitochondrial and nuclear SNP genotypes for all individuals used in this study.
