# Supplementary figures and images for: Steep, coincident, and concordant clines in mitochondrial and nuclear‐encoded genes in a hybrid zone between subspecies of Atlantic killifish, Fundulus heteroclitus
Source: Ecol Evol. 2016 Jul 22;6(16):5771–87. doi: 10.1002/ece3.2324 (PMC4983590; doi:10.1002/ece3.2324)

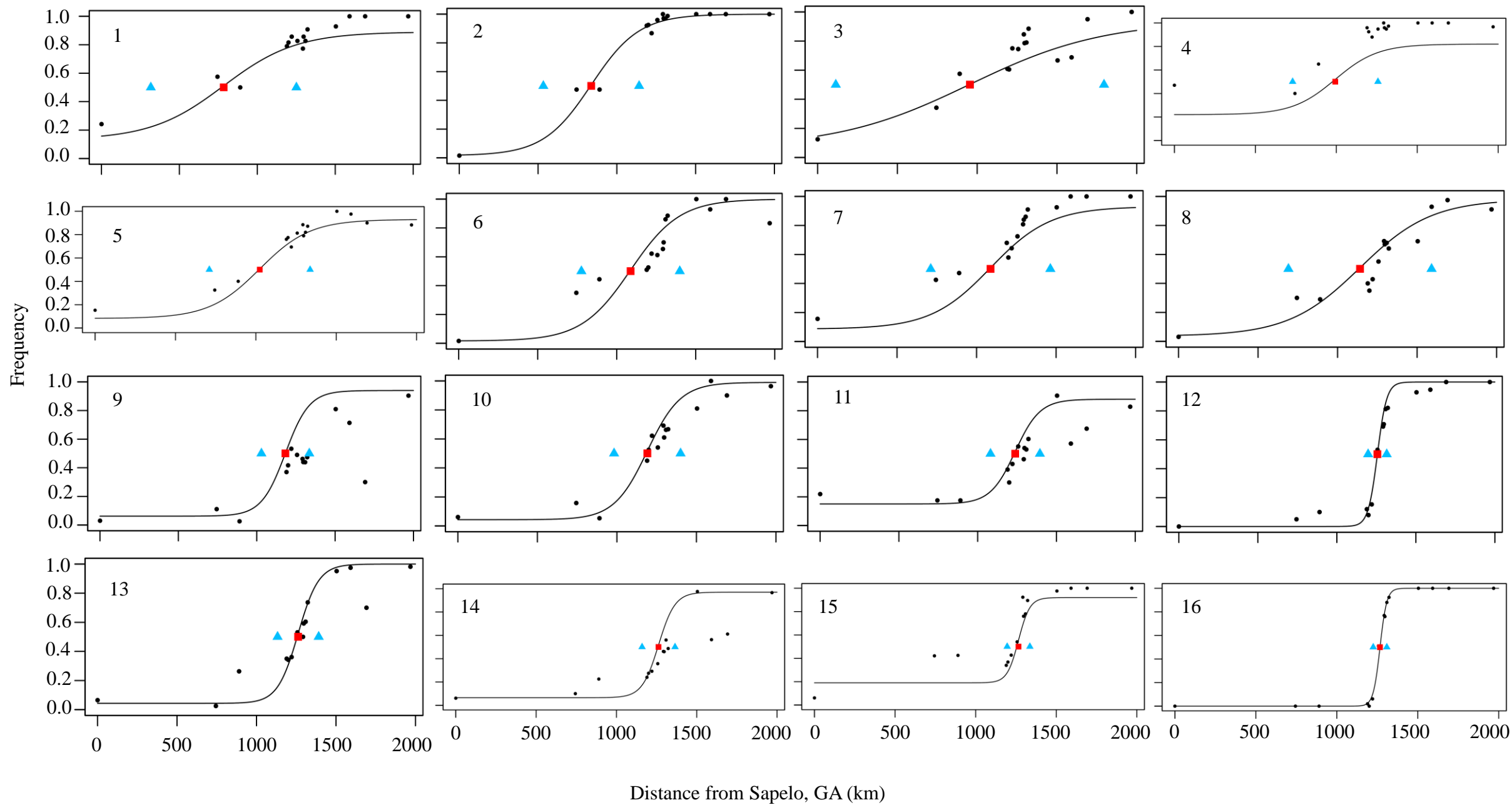

Frequency

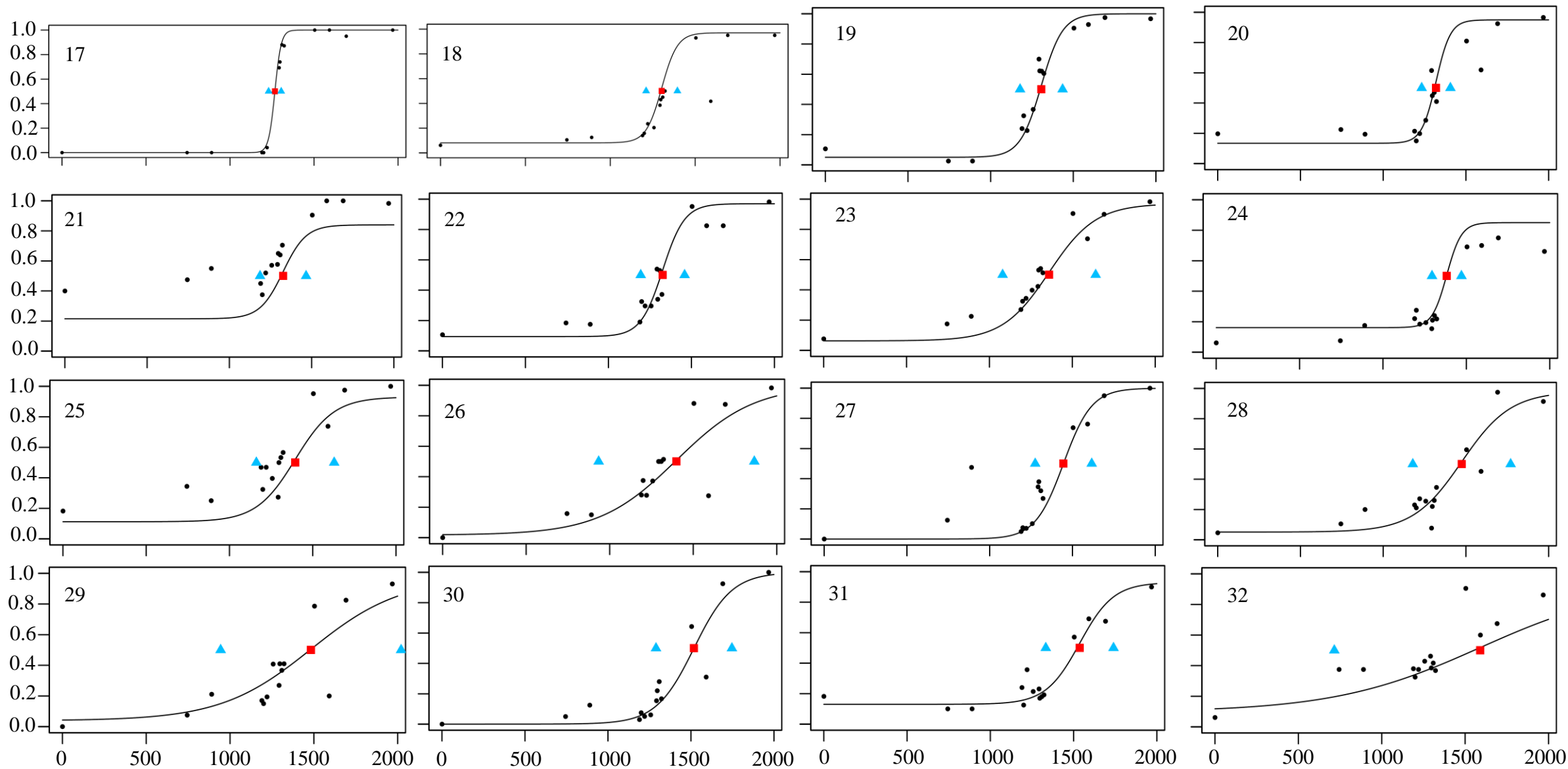

Distance from Sapelo, GA (km)

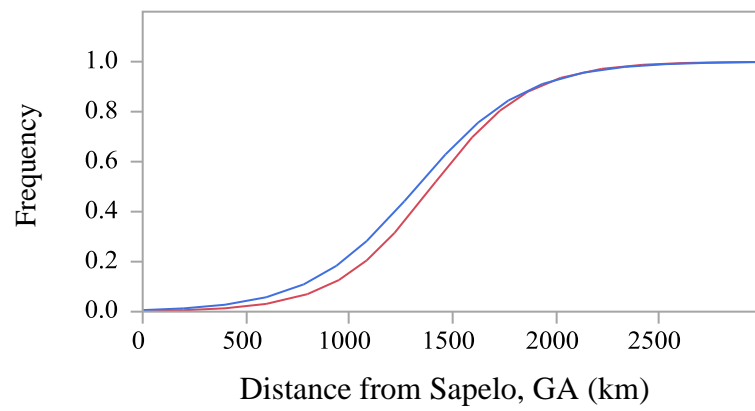

Supplement: Supplementary file 1 — Figure S1. Clines in allele frequency for all SNP loci included in this study. Figure S2. Cline in Fundulus heteroclitus Ldh‐B allele frequency from the current data set (shown in red) and data from Powers and Place (1978) shown in blue. [file ECE3-6-5771-s001.pdf]
