# Supplementary material for: Steep, coincident, and concordant clines in mitochondrial and nuclear‐encoded genes in a hybrid zone between subspecies of Atlantic killifish, Fundulus heteroclitus
Source: Ecol Evol. 2016 Jul 22;6(16):5771–87. doi: 10.1002/ece3.2324 (PMC4983590; doi:10.1002/ece3.2324)
Supplement: Supplementary file 3 — Table S2. Summary of locus pairs in significant linkage disequilibrium after FDR‐adjustment at each location. Table S3. Number of loci showing deviations from HWE, LD or cytonuclear disequilibrium prior to FDR correction. Table S4. Summary of locus pairs in significant linkage disequilibrium at each location. Table S5. F IS values for loci that deviated significantly from HWE. Table S6. Four cline shape parameters as predicted by ClineFit, arranged by order of increasing centre (as in Fig 3). [file ECE3-6-5771-s003.docx]

Table S2. Summary of locus pairs in significant linkage disequilibrium after FDR-adjustment at each location.

| Locus A | Locus B | Rij | p-value |
| --- | --- | --- | --- |
| **Wiscasset, ME** |  |  |  |
| Locus 9: 1176 | Locus 26:LDHB1033 | 0.78021 | 0.0001 |
| Locus 9: 1176 | Locus 23: LDHB654 | 0.78021 | 0.0001 |
| Locus 26: LDHB1033 | Locus 23: LDHB654 | 0.99999 | 0.0001 |
| Locus 28:Cytochrome p450 | Locus 10: 60S ribosomal protein L6 | 0.70432 | 0.0003 |
| Locus 20: WAP | Locus 4: Translationally controlled tumor protein | 0.71916 | 0.0001 |
| Locus 29: 14-3-3 zeta | Locus 14: 60S ribosomal protein L35 | 0.70471 | 0.0001 |
| Locus 14: 60S ribosomal protein L35 | Locus 4: Translationally controlled tumor protein | 0.71916 | 0.0001 |
| **Sandwich, MA** |  |  |  |
| Locus 19: HDDC2 | Locus 28: Cytochrome p450 | 0.99999 | 0.0001 |
| Locus 26:LDHB1033 | Locus 23:LDHB654 | 0.91161 | 0.0001 |

Table S3: Number of loci showing deviations from HWE, LD or cytonuclear disequilibrium prior to FDR correction. Positive Rij = number of pairs of nuclear loci in significant linkage disequilibrium such that alleles from like parental type are more often together: Negative Rij = number of pairs of nuclear loci in significant linkage disequilibrium such that alleles from unlike parental types are more often found together.

| Sampling location | Number of loci exhibiting heterozygote deficit | Number of loci exhibiting heterozygote excess | Number of loci with positive Rij | Number of loci with negative Rij | Number of loci exhibiting cytonuclear disequilibrium |
| --- | --- | --- | --- | --- | --- |
| 2. Wiscasset, ME | 0 | 0 | 22 | 2 | 0 |
| 6. Sandwich, MA | 0 | 0 | 21 | 3 | 0 |
| 8. Point Judith, RI | 0 | 0 | 17 | 8 | 0 |
| 9. Clinton, CT | 0 | 0 | 20 | 3 | 0 |
| 13. Cheesequake, NJ | 3 | 1 | 11 | 7 | 1 |
| 15. Belford, NJ | 6 | 1 | 25 | 7 | 2 |
| 16. Sandy Hook, NJ | 2 | 0 | 18 | 5 | 2 |
| 17. Navesink, NJ | 0 | 0 | 17 | 10 | 0 |
| 19. Metedeconk, NJ | 5 | 0 | 20 | 9 | 3 |
| 20. Laurel, NJ | 4 | 0 | 12 | 12 | 0 |
| 21. Tuckerton, NJ | 0 | 0 | 18 | 16 | 0 |
| 22. RUMFS, NJ | 1 | 0 | 14 | 8 | 0 |
| 28. Magotha, VA | 0 | 0 | 12 | 9 | 0 |
| 30. Manteo, NC | 1 | 0 | 30 | 11 | 0 |
| 35. Sapelo Island, GA | 0 | 0 | 21 | 8 | 0 |

Table S4 Summary of locus pairs in significant linkage disequilibrium at each location. Location names are in bold. Values marked with an * are significant after FDR-adjustment.

| Locus 1 | Locus 2 | Rij | p-value |
| --- | --- | --- | --- |
| **Wiscasset, ME** |  |  |  |
| 1176 | LDHB1033 | 0.78021 | 0.0001* |
| 1176 | LDHB654 | 0.78021 | 0.0001* |
| 1176 | nucleotide_diphosphate_kinase2 | 0.4293 | 0.0286 |
| 1176 | SLC25A3 | 0.50317 | 0.0103 |
| HDDC2 | Chymotrypsin-C | 0.40106 | 0.0338 |
| LDHB1033 | LDHB654 | 0.99999 | 0.0001* |
| LDHB1033 | Chymotrypsin-C | 0.40106 | 0.0338 |
| LDHB1033 | SLC25A3 | 0.5763 | 0.0016 |
| LDHB 654 | Chymotrypsin-C | 0.40106 | 0.0338 |
| LDHB 654 | SLC25A3 | 0.5763 | 0.0016 |
| Cytochrome p450 | 60S_ribosomal_protein_L6 | 0.70432 | 0.0003* |
| Cytochrome p450 | nucleotide_diphosphate_kinase2 | 0.45876 | 0.0135 |
| Cytochrome p450 | Parvalbumin | 0.44689 | 0.018 |
| Myoglobin | nucleotide_diphosphate_kinase2 | 0.428025 | 0.0235 |
| Chymotrypsin-C | Guanine nucleotide binding protein | -0.445 | 0.0185 |
| Chymotrypsin-C | 14-3-3 ZETA | -0.39367 | 0.0408 |
| Guanine nucleotide binding protein | nucleotide_diphosphate_kinase2 | 0.36574 | 0.0451 |
| Nucleotide diphosphate kinase2 | Tropomyosin | 0.38216 | 0.0432 |
| GAPDH | 14-3-3 ZETA | 0.48933 | 0.0084 |
| WAP | 60S_ribosomal_protein_L35 | 0.47863 | 0.0113 |
| WAP | Translationally controlled tumor protein | 0.71916 | 0.0001* |
| 14-3-3 ZETA | 60S_ribosomal_protein_L35 | 0.70471 | 0.0001* |
| 14-3-3 ZETA | Translationally controlled tumor protein | 0.48933 | 0.0084 |
| 60S_ribosomal_protein_L35 | Translationally controlled tumor protein | 0.71916 | 0.0001* |
| **Sandwich, MA** |  |  |  |
| 1173 | 14-3-3 ZETA | -0.68451 | 0.0022 |
| 1176 | Myoglobin | 0.49536 | 0.0267 |
| 1176 | Parvalbumin | -0.44176 | 0.0482 |
| HDDC2 | LDHB654 | 0.48298 | 0.0308 |
| HDDC2 | Cytochrome p450 | 0.99999 | 0.0001* |
| HDDC2 | ribsomal_protein | 0.48298 | 0.0308 |
| HDDC2 | SLC25A3 | 0.72447 | 0.0012 |
| LDHB1033 | LDHB654 | 0.91161 | 0.0001* |
| LDHB1033 | ribosomal_protein | 0.60774 | 0.0066 |
| LDHB1033 | SLC25A3 | 0.60774 | 0.0066 |
| LDHB 654 | Cytochrome p450 | 0.48298 | 0.0308 |
| LDHB 654 | nucleotide_diphosphate_kinase1 | 0.48298 | 0.0308 |
| LDHB 654 | SLC25A3 | 0.70175 | 0.0017 |
| Cytochrome p450 | ribosomal_protein | 0.48298 | 0.0308 |
| Cytochrome p450 | SLC25A3 | 0.72447 | 0.0012 |
| Myoglobin | Parvalbumin | -0.52459 | 0.019 |
| Myoglobin | Hemoglobin alpha | 0.54157 | 0.0154 |
| Myoglobin | Tropomyosin | 0.52988 | 0.0178 |
| Chymotrypsin-C | Hemoglobin alpha | 0.52306 | 0.0193 |
| Parvalbumin | WAP | 0.46214 | 0.0388 |
| Hemoglobin beta | 60S_ribosomal_protein_L35 | 0.50737 | 0.0313 |
| Hemoglobin alpha | Tropomyosin | 0.47817 | 0.0325 |
| Tropomyosin | ribosomal_protein | 0.44138 | 0.0484 |
| **Point Judith, RI** |  |  |  |
| 1173 | Guanine nucleotide binding protein | -0.5147 | 0.0462 |
| 1173 | Titin cap | 0.55759 | 0.0308 |
| 1176 | Chymotrypsin-C | 0.54226 | 0.0153 |
| 1176 | Parvalbumin | 0.52893 | 0.0154 |
| HDDC2 | Actin binding LIM protein family 3 | 0.46569 | 0.0424 |
| HDDC2 | Guanine nucleotide binding protein | 0.64167 | 0.0033 |
| Actin binding LIM protein family 3 | ribosomal_protein | 0.72536 | 0.0016 |
| LDHB1033 | LDHB654 | 0.5348 | 0.0168 |
| LDHB 654 | Hemoglobin alpha | 0.43796 | 0.0447 |
| LDHB 654 | SLC25A3 | -0.86155 | 0.0003 |
| Myoglobin | nucleotide_diphosphate_kinase1 | 0.53999 | 0.0157 |
| Myoglobin | 60S_ribosomal_protein_L35 | 0.63445 | 0.014 |
| Chymotrypsin-C | ribosomal_protein_S2 | -0.5727 | 0.0266 |
| Chymotrypsin-C | nucleotide_diphosphate_kinase2 | 0.45884 | 0.0402 |
| Chymotrypsin-C | Parvalbumin | 0.52438 | 0.019 |
| Chymotrypsin-C | Titin cap | 0.47607 | 0.0332 |
| ribosomal_protein_S2 | Parvalbumin | -0.75899 | 0.0024 |
| ribosomal_protein_S2 | 60S_ribosomal_protein_L35 | 0.6029 | 0.0159 |
| Nucleotide diphosphate kinase1 | Hemoglobin beta | -0.43743 | 0.045 |
| Guanine nucleotide binding protein | WAP | 0.51718 | 0.0178 |
| Nucleotide diphosphate kinase2 | ribosomal_protein | 0.44896 | 0.0396 |
| Parvalbumin | Tropomyosin | -0.44819 | 0.045 |
| Hemoglobin beta | Hemoglobin alpha | 0.49418 | 0.0235 |
| Hemoglobin beta | 14-3-3 ZETA | -0.51828 | 0.0205 |
| 60S_ribosomal_protein_L35 | SLC25A3 | -0.60985 | 0.0279 |
| **Clinton, CT** |  |  |  |
| 1176 | 60S_ribosomal_protein_L35 | 0.5822 | 0.0076 |
| HDDC2 | nucleotide_diphosphate_kinase2 | -0.5528 | 0.0113 |
| HDDC2 | GAPDH | 0.72574 | 0.0009 |
| Actin binding LIM protein family 3 | 40S_ribsomal_protein_S17 | 0.57511 | 0.0084 |
| Actin binding LIM protein family 3 | 60S_ribosomal_protein_L35 | 0.57511 | 0.0084 |
| LDHB1033 | LDHB654 | 0.97988 | 0.0001 |
| LDHB1033 | Atrial Natriuretic peptide | 0.62095 | 0.0044 |
| LDHB1033 | SLC25A3 | 0.89261 | 0.0001 |
| LDHB 654 | Atrial natriuretic peptide | 0.51983 | 0.0172 |
| LDHB 654 | SLC25A3 | 0.88371 | 0.0001 |
| Cytochrome p450 | GAPDH | 0.48074 | 0.0276 |
| Myoglobin | ribosomal_protein_S2 | 0.44191 | 0.0429 |
| Myoglobin | nucleotide_diphosphate_kinase2 | -0.52199 | 0.0168 |
| Chymotrypsin-C | 14-3-3 ZETA | 0.65986 | 0.0025 |
| ribosomal_protein_S2 | GAPDH | 0.46248 | 0.0341 |
| ribosomal_protein_S2 | Parvalbumin | 0.46248 | 0.0341 |
| ribosomal_protein_S2 | NACA | 0.55613 | 0.0129 |
| Nucleotide diphosphate kinase1 | 60S_ribosomal_protein_L35 | 0.72366 | 0.0009 |
| Nucleotide diphosphate kinase2 | Parvalbumin | -0.5528 | 0.0113 |
| Hemoglobin beta | Hemoglobin alpha | 0.64491 | 0.0031 |
| Hemoglobin alpha | WAP | 0.71189 | 0.0011 |
| WAP | 14-3-3 ZETA | 0.53214 | 0.0147 |
| 1173 | ribosomal_protein_S2 | 0.54798 | 0.012 |
| **Cheesequake, NJ** |  |  |  |
| 1173 | nucleotide_diphosphate_kinase1 | 0.3224 | 0.0499 |
| 1176 | GAPDH | 0.32492 | 0.0424 |
| 1176 | 60S_ribosomal_protein_L35 | 0.33277 | 0.0377 |
| HDDC2 | Parvalbumin | -0.38408 | 0.0179 |
| LDHB1033 | LDHB654 | 0.68644 | 0.0001 |
| LDHB1033 | SLC25A3 | 0.44928 | 0.0063 |
| LDHB 654 | SLC25A3 | 0.46471 | 0.0042 |
| Cytochrome p450 | Translationally controlled tumor protein | -0.52323 | 0.0015 |
| Myoglobin | Tropomyosin | 0.38416 | 0.0179 |
| Chymotrypsin-C | ribosomal_protein_S2 | -0.43138 | 0.0071 |
| Chymotrypsin-C | NACA | -0.34254 | 0.0324 |
| Chymotrypsinogen | Parvalbumin | 0.33067 | 0.0389 |
| ribosomal_protein_S2 | 40S_ribsomal_protein_S17 | 0.37658 | 0.0203 |
| Guanine nucleotide binding protein | Tropomyosin | -0.38113 | 0.0173 |
| Nucleotide diphosphate kinase2 | ribosomal_protein | -0.31443 | 0.0496 |
| Parvalbumin | 40S_ribsomal_protein_S17 | -0.35814 | 0.0273 |
| Hemoglobin beta | Hemoglobin alpha | 0.7798 | 0.0001 |
| Hemoglobin alpha | 40S_ribsomal_protein_S17 | 0.41025 | 0.0114 |
| **Belford, NJ** |  |  |  |
| 1173 | LDHB654 | 0.2864 | 0.0496 |
| 1173 | Guanine nucleotide binding protein | 0.29335 | 0.0421 |
| 1173 | Guanine nucleotide binding protein | 0.3537 | 0.0153 |
| 1176 | HDDC2 | 0.35757 | 0.0165 |
| HDDC2 | Chymotrypsin-C | 0.3685 | 0.0124 |
| Actin binding LIM protein family 3 | Chymotrypsinogen | 0.47068 | 0.0011 |
| LDHB1033 | LDHB654 | 0.69699 | 0.0001 |
| LDHB1033 | Myoglobin | -0.33427 | 0.0249 |
| LDHB1033 | Guanine nucleotide binding protein | 0.36356 | 0.0127 |
| LDHB1033 | GAPDH | 0.3898 | 0.0075 |
| LDHB1033 | SLC25A3 | 0.31159 | 0.0327 |
| LDHB 654 | Guanine nucleotide binding protein | 0.2881 | 0.0437 |
| LDHB 654 | GAPDH | 0.29005 | 0.0423 |
| LDHB 654 | SLC25A3 | 0.34204 | 0.0167 |
| Cytochrome p450 | nucleotide_diphosphate_kinase1 | 0.30764 | 0.039 |
| Cytochrome p450 | 14-3-3 ZETA | -0.31634 | 0.0268 |
| Chymotrypsin-C | Hemoglobin beta | 0.27935 | 0.0482 |
| Chymotrypsinogen | NACA | 0.31621 | 0.0254 |
| Chymotrypsinogen | 40S_ribsomal_protein_S17 | 0.39961 | 0.0047 |
| Guanine nucleotide binding protein | GAPDH | 0.36988 | 0.0089 |
| Guanine nucleotide binding protein | WAP | -0.31938 | 0.0239 |
| Guanine nucleotide binding protein | ribosomal_protein | -0.30108 | 0.0333 |
| Guanine nucleotide binding protein | SLC25A3 | 0.30797 | 0.0294 |
| GAPDH | Translationally controlled tumor protein | -0.28958 | 0.0448 |
| Parvalbumin | Titin cap | 0.2824 | 0.0458 |
| Titin cap | WAP | -0.31041 | 0.0282 |
| Hemoglobin beta | Hemoglobin alpha | 0.81576 | 0.0001 |
| NACA | 40S_ribsomal_protein_S17 | 0.36343 | 0.0102 |
| Hemoglobin alpha | 40S_ribsomal_protein_S17 | 0.28693 | 0.0425 |
| Tropomyosin | 14-3-3 ZETA | -0.29609 | 0.0382 |
| Tropomyosin | ribosomal_protein | 0.30409 | 0.0315 |
| WAP | 14-3-3 ZETA | 0.29855 | 0.0366 |
| **Sandy Hook, NJ** |  |  |  |
| 1173 | Actin binding LIM protein family 3 | -0.30378 | 0.0394 |
| 1173 | GAPDH | 0.35305 | 0.0144 |
| 1176 | LDHB1033 | 0.34948 | 0.0135 |
| 1176 | ribosomal_protein_S2 | 0.29511 | 0.0369 |
| HDDC2 | Actin binding LIM protein family 3 | 0.30086 | 0.0391 |
| HDDC2 | hemoglobin_a2 | 0.29823 | 0.0368 |
| Actin binding LIM protein family 3 | LDHB1033 | 0.30391 | 0.0352 |
| LDHB1033 | LDHB654 | 0.70043 | 0.0001 |
| LDHB1033 | 14-3-3 ZETA | -0.30484 | 0.0311 |
| LDHB1033 | SLC25A3 | 0.32589 | 0.0212 |
| LDHB 654 | SLC25A3 | 0.2936 | 0.0399 |
| Cytochrome p450 | Chymotrypsin-C | 0.30136 | 0.0331 |
| Myoglobin | Guanine nucleotide binding protein | 0.34173 | 0.0168 |
| Myoglobin | Parvalbumin | 0.30442 | 0.0331 |
| Chymotrypsin-C | 60S_ribosomal_protein_L6 | 0.34871 | 0.0137 |
| Chymotrypsinogen | Atrial natriuretic peptide | 0.28774 | 0.044 |
| Nucleotide diphosphate kinase1 | Hemoglobin alpha | -0.44051 | 0.0028 |
| Parvalbumin | WAP | 0.32148 | 0.0244 |
| Titin cap | 40S_ribsomal_protein_S17 | -0.28669 | 0.0426 |
| Hemoglobin beta | Hemoglobin alpha | 0.67494 | 0.0001 |
| Hemoglobin beta | 40S_ribsomal_protein_S17 | 0.2962 | 0.0362 |
| NACA | 40S_ribsomal_protein_S17 | 0.28187 | 0.0463 |
| Tropomyosin | 14-3-3 ZETA | -0.28121 | 0.0468 |
| **Navesink, NJ** |  |  |  |
| 1173 | ribosomal_protein_S2 | 0.61317 | 0.027 |
| 1173 | Guanine nucleotide binding protein | 0.60096 | 0.0462 |
| 1176 | LDHB1033 | 0.6222 | 0.0311 |
| 1176 | LDHB654 | 0.58431 | 0.0351 |
| 1176 | Myoglobin | -0.58227 | 0.0358 |
| HDDC2 | LDHB654 | 0.7817 | 0.0134 |
| HDDC2 | Cytochrome p450 | -0.75378 | 0.0171 |
| HDDC2 | NACA | 0.80416 | 0.011 |
| Actin binding LIM protein family 3 | Parvalbumin | 0.54851 | 0.048 |
| Actin binding LIM protein family 3 | Hemoglobin alpha | 0.58262 | 0.0357 |
| Actin binding LIM protein family 3 | 40S_ribsomal_protein_S17 | 0.76544 | 0.0058 |
| Actin binding LIM protein family 3 | 60S_ribosomal_protein_L35 | 0.5992 | 0.0307 |
| LDHB1033 | LDHB654 | 0.84501 | 0.0034 |
| LDHB1033 | Cytochrome p450 | -0.56971 | 0.0484 |
| LDHB1033 |  | -0.57854 | 0.0451 |
| LDHB1033 | NACA | 0.73798 | 0.0106 |
| LDHB 654 | NACA | 0.82447 | 0.003 |
| Chymotrypsin-C | 60S_ribosomal_protein_L6 | -0.73342 | 0.0082 |
| ribosomal_protein_S2 | ribosomal_protein | 0.67822 | 0.0145 |
| 60S_ribosomal_protein_L6 | 14-3-3 ZETA | 0.59142 | 0.033 |
| Nucleotide diphosphate kinase1 | Parvalbumin | -0.7379 | 0.0144 |
| Nucleotide diphosphate kinase2 | ribosomal_protein | -0.64091 | 0.0208 |
| Nucleotide diphosphate kinase2 | 60S_ribosomal_protein_L35 | -0.58147 | 0.036 |
| Atrial natriuretic peptide | Parvalbumin | -0.75889 | 0.0118 |
| GAPDH | Tropomyosin | -0.61664 | 0.0262 |
| GAPDH | ribosomal_protein | 0.63292 | 0.0225 |
| Hemoglobin beta | Hemoglobin alpha | 0.66499 | 0.0165 |
| **Metedeconk, NJ** |  |  |  |
| 1176 | SLC25A3 | 0.44192 | 0.0022 |
| HDDC2 | 60S_ribosomal_protein_L35 | 0.33405 | 0.0194 |
| Actin binding LIM protein family 3 | Chymotrypsin-C | -0.28323 | 0.0497 |
| LDHB1033 | LDHB654 | 0.49927 | 0.0006 |
| LDHB1033 | ribosomal_protein | 0.36676 | 0.0129 |
| LDHB 654 | 60S_ribosomal_protein_L6 | -0.37738 | 0.0083 |
| LDHB 654 | Atrial natriuretic peptide | -0.29352 | 0.0399 |
| LDHB 654 | 14-3-3 ZETA | 0.4709 | 0.001 |
| LDHB 654 | SLC25A3 | 0.39704 | 0.0054 |
| Cytochrome p450 | 60S_ribosomal_protein_L6 | 0.280255 | 0.0495 |
| Cytochrome p450 | GAPDH | 0.33053 | 0.0207 |
| Cytochrome p450 | 14-3-3 ZETA | -0.37646 | 0.0084 |
| Myoglobin | Translationally controlled tumor protein | 0.33648 | 0.0211 |
| Chymotrypsin-C | ribosomal_protein_S2 | 0.31421 | 0.0312 |
| Chymotrypsin-C | NACA | 0.35522 | 0.0129 |
| Chymotrypsin-C | WAP | -0.31759 | 0.0262 |
| ribosomal_protein_S2 | GAPDH | 0.30811 | 0.0347 |
| 60S_ribosomal_protein_L6 | Titin cap | 0.34429 | 0.016 |
| 60S_ribosomal_protein_L6 | Translationally controlled tumor protein | 0.33566 | 0.02 |
| Nucleotide diphosphate kinase1 | Translationally controlled tumor protein | 0.2942 | 0.0437 |
| Guanine nucleotide binding protein | Translationally controlled tumor protein | -0.31445 | 0.0294 |
| Nucleotide diphosphate kinase2 | Parvalbumin | -0.28353 | 0.0472 |
| Hemoglobin beta | Hemoglobin alpha | 0.43767 | 0.0022 |
| Hemoglobin beta | SLC25A3 | -0.29289 | 0.0403 |
| NACA | 60S_ribosomal_protein_L35 | -0.32866 | 0.0214 |
| Hemoglobin alpha | WAP | 0.30413 | 0.0333 |
| Tropomyosin | ribosomal_protein | 0.49331 | 0.0006 |
| Tropomyosin | Translationally controlled tumor protein | 0.28858 | 0.0456 |
| 14-3-3 ZETA | SLC25A3 | 0.33572 | 0.0188 |
| **Laurel, NJ** |  |  |  |
| HDDC2 | Guanine nucleotide binding protein | -0.33475 | 0.0264 |
| HDDC2 | Titin cap | 0.30604 | 0.0424 |
| Actin binding LIM protein family 3 | Myoglobin | 0.43455 | 0.0044 |
| Actin binding LIM protein family 3 | ribosomal_protein_S2 | -0.3333 | 0.0209 |
| Actin binding LIM protein family 3 | Atrial natriuretic peptide | -0.32282 | 0.0238 |
| Actin binding LIM protein family 3 | Titin cap | 0.29469 | 0.0391 |
| LDHB1033 | LDHB654 | 0.56767 | 0.0002 |
| LDHB 654 | Myoglobin | -0.33486 | 0.03 |
| LDHB 654 | 60S_ribosomal_protein_L6 | 0.29476 | 0.0411 |
| Cytochrome p450 | 60S_ribosomal_protein_L35 | 0.32228 | 0.0271 |
| Myoglobin | Atrial natriuretic peptide | -0.36728 | 0.016 |
| Myoglobin | Hemoglobin alpha | -0.34768 | 0.0226 |
| Chymotrypsin-C | Hemoglobin beta | 0.32314 | 0.0237 |
| Chymotrypsinogen | Guanine nucleotide binding protein | -0.30124 | 0.035 |
| Nucleotide diphosphate kinase1 | Translationally controlled tumor protein | -0.35958 | 0.0147 |
| Guanine nucleotide binding protein | Parvalbumin | -0.41321 | 0.0038 |
| Nucleotide diphosphate kinase2 | Tropomyosin | 0.33976 | 0.0174 |
| GAPDH | Hemoglobin alpha | -0.33039 | 0.0207 |
| Hemoglobin beta | Hemoglobin alpha | 0.74886 | 0.0001 |
| Hemoglobin alpha | Tropomyosin | 0.28514 | 0.0459 |
| Hemoglobin alpha | Translationally controlled tumor protein | -0.42127 | 0.0043 |
| Tropomyosin | WAP | 0.29655 | 0.0399 |
| Tropomyosin | Translationally controlled tumor protein | -0.36572 | 0.0131 |
| 40S_ribsomal_protein_S17 | Translationally controlled tumor protein | 0.30405 | 0.0414 |
| **Tuckerton, NJ** |  |  |  |
| 1173 | 14-3-3 ZETA | -0.45859 | 0.0403 |
| 1176 | HDDC2 | 0.46712 | 0.0475 |
| 1176 | ribosomal_protein_S2 | 0.59587 | 0.014 |
| 1176 | nucleotide_diphosphate_kinase2 | -0.79182 | 0.0011 |
| 1176 | SLC25A3 | -0.67252 | 0.0056 |
| HDDC2 | nucleotide_diphosphate_kinase2 | -0.7199 | 0.0017 |
| HDDC2 | SLC25A3 | -0.45017 | 0.0497 |
| Actin binding LIM protein family 3 | LDHB1033 | 0.45426 | 0.0477 |
| Actin binding LIM protein family 3 | WAP | 0.69256 | 0.0025 |
| LDHB1033 | LDHB654 | 0.60527 | 0.0068 |
| LDHB1033 | 60S_ribosomal_protein_L6 | -0.58851 | 0.0085 |
| LDHB1033 | WAP | 0.64749 | 0.0038 |
| LDHB1033 | 60S_ribosomal_protein_L35 | 0.5682 | 0.0111 |
| LDHB 654 | Guanine nucleotide binding protein | -0.5857 | 0.0107 |
| LDHB 654 | Atrial natriuretic peptide | 0.49108 | 0.0323 |
| LDHB 654 | Parvalbumin | 0.50968 | 0.0226 |
| LDHB 654 | Titin cap | -0.46214 | 0.0388 |
| LDHB 654 | 14-3-3 ZETA | -0.50941 | 0.0227 |
| LDHB 654 | 40S_ribsomal_protein_S17 | 0.486 | 0.0341 |
| LDHB 654 | Translationally controlled tumor protein | -0.46214 | 0.0388 |
| Cytochrome p450 | SLC25A3 | -0.59726 | 0.0113 |
| Myoglobin | ribosomal_protein_S2 | 0.51665 | 0.0284 |
| Myoglobin | 40S_ribsomal_protein_S17 | 0.62859 | 0.0077 |
| Chymotrypsin-C | Hemoglobin beta | 0.5876 | 0.0086 |
| Chymotrypsin-C | Hemoglobin alpha | 0.5876 | 0.0086 |
| Chymotrypsin-C | 60S_ribosomal_protein_L35 | -0.54737 | 0.0144 |
| ribosomal_protein_S2 | 40S_ribsomal_protein_S17 | 0.62615 | 0.0079 |
| Nucleotide diphosphate kinase1 | Parvalbumin | 0.54268 | 0.0253 |
| Nucleotide diphosphate kinase1 | Titin cap | -0.64214 | 0.0081 |
| Guanine nucleotide binding protein | Atrial natriuretic peptide | -0.64305 | 0.0064 |
| Guanine nucleotide binding protein | Parvalbumin | -0.46821 | 0.0413 |
| Guanine nucleotide binding protein | 40S_ribsomal_protein_S17 | -0.46564 | 0.0482 |
| Nucleotide diphosphate kinase2 | GAPDH | 0.51538 | 0.0212 |
| Atrial natriuretic peptide | SLC25A3 | 0.45447 | 0.0476 |
| Parvalbumin | 14-3-3 ZETA | -0.45018 | 0.0441 |
| Titin cap | Tropomyosin | 0.49722 | 0.0262 |
| Hemoglobin beta | Hemoglobin alpha | 0.99999 | 0.0001 |
| ribosomal_protein | SLC25A3 | -0.50043 | 0.0292 |
| 40S_ribsomal_protein_S17 | Translationally controlled tumor protein | -0.59529 | 0.0095 |
| **RUMFS, NJ** |  |  |  |
| 1173 | GAPDH | 0.37696 | 0.0106 |
| 1176 | ribosomal_protein_S2 | 0.32183 | 0.0291 |
| 1176 | 14-3-3 ZETA | -0.30852 | 0.0291 |
| Actin binding LIM protein family 3 | Guanine nucleotide binding protein | 0.32908 | 0.02 |
| LDHB1033 | LDHB654 | 0.36549 | 0.0098 |
| LDHB1033 | WAP | 0.43042 | 0.0026 |
| LDHB 654 | Chymotrypsinogen | -0.28182 | 0.0463 |
| Myoglobin | ribosomal_protein | 0.29024 | 0.0401 |
| Myoglobin | 60S_ribosomal_protein_L35 | 0.32855 | 0.0202 |
| Chymotrypsin-C | Titin cap | 0.39851 | 0.0048 |
| 60S_ribosomal_protein_L6 | nucleotide_diphosphate_kinase1 | 0.280233 | 0.0475 |
| Nucleotide diphosphate kinase1 | SLC25A3 | -0.29943 | 0.038 |
| Guanine nucleotide binding protein | Parvalbumin | -0.2911 | 0.0396 |
| Guanine nucleotide binding protein | Titin cap | 0.28137 | 0.0466 |
| Nucleotide diphosphate kinase2 | Atrial natriuretic peptide | 0.41636 | 0.0032 |
| Atrial natriuretic peptide | Translationally controlled tumor protein | 0.28744 | 0.0442 |
| Parvalbumin | hemoglobin_a2 | -0.28137 | 0.0466 |
| Parvalbumin | 14-3-3 ZETA | -0.36905 | 0.0091 |
| Titin cap | Tropomyosin | -0.30475 | 0.0312 |
| Hemoglobin beta | Hemoglobin alpha | 0.7734 | 0.0001 |
| Hemoglobin beta | 60S_ribosomal_protein_L35 | 0.38319 | 0.0067 |
| **Magotha, VA** |  |  |  |
| 1173 | LDHB654 | -0.51363 | 0.0216 |
| 1173 | ribsomal_protein | 0.44457 | 0.0468 |
| 1173 | 40S_ribsomal_protein_S17 | -0.47499 | 0.0337 |
| 1173 | SLC25A3 | 0.56577 | 0.0114 |
| 1176 | Actin binding LIM protein family 3 | 0.48179 | 0.0357 |
| Actin binding LIM protein family 3 | nucleotide_diphosphate_kinase2 | 0.5677 | 0.0111 |
| Actin binding LIM protein family 3 | 60S_ribosomal_protein_L35 | -0.47607 | 0.0332 |
| LDHB1033 | Titin cap | -0.45511 | 0.0473 |
| LDHB1033 | NACA | -0.50293 | 0.0329 |
| LDHB 654 | GAPDH | 0.59901 | 0.0074 |
| LDHB 654 | 60S_ribosomal_protein_L35 | -0.48235 | 0.031 |
| Cytochrome p450 | Guanine nucleotide binding protein | 0.56968 | 0.0157 |
| Cytochrome p450 | nucleotide_diphosphate_kinase2 | 0.84029 | 0.0002 |
| Chymotrypsin-C | 60S_ribosomal_protein_L6 | 0.73921 | 0.0013 |
| Chymotrypsin-C | nucleotide_diphosphate_kinase1 | 0.45984 | 0.0397 |
| Chymotrypsinogen | 14-3-3 ZETA | 0.59881 | 0.009 |
| ribosomal_protein_S2 | NACA | 0.54999 | 0.0196 |
| ribosomal_protein_S2 | ribosomal_protein | -0.58228 | 0.0092 |
| ribosomal_protein_S2 | SLC25A3 | -0.6769 | 0.0025 |
| Titin cap | Hemoglobin alpha | -0.78864 | 0.0006 |
| Hemoglobin alpha | SLC25A3 | 0.46393 | 0.038 |
| **Manteo, NC** |  |  |  |
| 1173 | NACA | 0.50995 | 0.0226 |
| 1173 | 40S_ribsomal_protein_S17 | 0.466 | 0.0422 |
| 1173 | 40S_ribsomal_protein_S17 | -0.45777 | 0.0406 |
| 1176 | Actin binding LIM protein family 3 | 0.70035 | 0.003 |
| 1176 | Myoglobin | 0.48043 | 0.0415 |
| 1176 | Chymotrypsin-C | 0.50621 | 0.0317 |
| 1176 | nucleotide_diphosphate_kinase2 | -0.54907 | 0.0198 |
| 1176 | GAPDH | -0.4906 | 0.0374 |
| 1176 | Hemoglobin beta | 0.48043 | 0.0415 |
| 1176 | Translationally controlled tumor protein | -0.47523 | 0.0438 |
| HDDC2 | Cytochrome p450 | 0.48179 | 0.0357 |
| HDDC2 | Guanine nucleotide binding protein | 0.49029 | 0.0283 |
| HDDC2 | nucleotide_diphosphate_kinase2 | 0.48298 | 0.0308 |
| Actin binding LIM protein family 3 | Myoglobin | 0.72447 | 0.0012 |
| Actin binding LIM protein family 3 | Atrial natriuretic peptide | -0.61714 | 0.0058 |
| LDHB1033 | Cytochrome p450 | 0.50918 | 0.0265 |
| LDHB1033 | Hemoglobin beta | 0.53293 | 0.0202 |
| LDHB 654 | Cytochrome p450 | 0.50918 | 0.0265 |
| LDHB 654 | nucleotide_diphosphate_kinase2 | 0.44138 | 0.0484 |
| Cytochrome p450 | ribosomal_protein_S2 | 0.57183 | 0.0153 |
| Cytochrome p450 | nucleotide_diphosphate_kinase2 | 0.5114 | 0.0258 |
| Myoglobin | Chymotrypsin-C | 0.57486 | 0.0101 |
| Chymotrypsin-C | Parvalbumin | 0.50225 | 0.0247 |
| Chymotrypsin-C | 14-3-3 ZETA | 0.479 | 0.0322 |
| Chymotrypsin-C | Translationally controlled tumor protein | -0.58959 | 0.0084 |
| Chymotrypsinogen | Translationally controlled tumor protein | 0.44138 | 0.0484 |
| 60S_ribosomal_protein_L6 | 40S_ribsomal_protein_S17 | 0.49936 | 0.0341 |
| 60S_ribosomal_protein_L6 | 60S_ribosomal_protein_L35 | 0.5114 | 0.0258 |
| Nucleotide diphosphate kinase1 | WAP | 0.60764 | 0.0151 |
| Nucleotide diphosphate kinase1 | ribosomal_protein | -0.65361 | 0.0089 |
| Guanine nucleotide binding protein | Tropomyosin | -0.55446 | 0.0157 |
| Guanine nucleotide binding protein | 40S_ribsomal_protein_S17 | 0.56678 | 0.0135 |
| GAPDH | Titin cap | -0.65873 | 0.0032 |
| Parvalbumin | hemoglobin_a2 | 0.47788 | 0.0326 |
| Parvalbumin | 14-3-3 ZETA | 0.56882 | 0.011 |
| Titin cap | SLC25A3 | -0.53416 | 0.0199 |
| Hemoglobin beta | 14-3-3 ZETA | 0.62529 | 0.0052 |
| Hemoglobin alpha | 14-3-3 ZETA | 0.57337 | 0.0103 |
| Hemoglobin alpha | 60S_ribosomal_protein_L35 | 0.60774 | 0.0066 |
| WAP | SLC25A3 | 0.56291 | 0.0141 |
| ribosomal_protein | 40S_ribsomal_protein_S17 | -0.53091 | 0.0207 |
| **Sapelo Island, GA** |  |  |  |
| 1173 | LDHB654 | -0.49086 | 0.0048 |
| 1173 | ribosomal_protein_S2 | 0.35487 | 0.0415 |
| 1173 | GAPDH | 0.37955 | 0.0376 |
| 1173 | ribsomal_protein | 0.51201 | 0.0033 |
| 1176 | HDDC2 | 0.60698 | 0.0005 |
| 1176 | Chymotrypsin-C | 0.49086 | 0.0048 |
| 1176 | Tropomyosin | 0.35134 | 0.0436 |
| 1176 | WAP | 0.42208 | 0.0153 |
| HDDC2 | Tropomyosin | 0.35805 | 0.0397 |
| LDHB 654 | GAPDH | -0.49458 | 0.0068 |
| Cytochrome p450 | Myoglobin | 0.54245 | 0.0025 |
| Cytochrome p450 | NACA | 0.64357 | 0.0003 |
| Myoglobin | Translationally controlled tumor protein | -0.44297 | 0.0137 |
| Chymotrypsin-C | 60S_ribosomal_protein_L35 | 0.4445 | 0.0107 |
| Chymotrypsin-C | SCL25A3 | 0.4445 | 0.0107 |
| Chymotrypsinogen | Tropomyosin | -0.35134 | 0.0436 |
| ribosomal_protein_S2 | Parvalbumin | -0.37943 | 0.0346 |
| 60S_ribosomal_protein_L6 | SLC25A3 | 0.35487 | 0.0415 |
| Nucleotide diphosphate kinase1 | Parvalbumin | 0.46988 | 0.0079 |
| Nucleotide diphosphate kinase1 | WAP | -0.35864 | 0.0394 |
| Nucleotide diphosphate kinase1 | 40S_ribsomal_protein_S17 | 0.73293 | 0.0001 |
| Nucleotide diphosphate kinase1 | 60S_ribosomal_protein_L35 | 0.41044 | 0.0184 |
| Guanine nucleotide binding protein | Titin cap | 0.99999 | 0.0001 |
| Guanine nucleotide binding protein | Tropomyosin | 0.3851 | 0.032 |
| Nucleotide diphosphate kinase2 | ribosomal_protein | 0.41912 | 0.0161 |
| GAPDH | SLC25A3 | 0.37955 | 0.0376 |
| Parvalbumin | ribosomal_protein | -0.37496 | 0.0339 |
| 40S_ribsomal_protein_S17 | 60S_ribosomal_protein_L35 | 0.4445 | 0.0107 |

Table S5. F_IS_ values for loci that deviated significantly from HWE. Locus numbers are as defined in Table 2. Positive values of F_IS_ indicate heterozygote deficit while negative values indicate heterozygote excess. F_IS_ values in bold are significant at α = 0.05; values significant after FDR-adjustment are marked with an asterisk (*). Hybrid zone locations emphasized with shading. NA = calculation not applicable because only a single allele was present among individuals from a particular sampling site.

| **Location** | **Locus Number** | | | | | | | | | | |
| --- | --- | --- | --- | --- | --- | --- | --- | --- | --- | --- | --- |
|  | 1 | 2 | 3 | 4 | 7 | 8 | 9 | 10 | 11 | 12 | 13 |
| 2. Wiscasset, ME | NA | NA | NA | 1.000 | NA | -0.080 | 0.353 | -0.019 | -0.191 | NA | NA |
| 6. Sandwich, MA | NA | NA | -0.027 | NA | NA | NA | -1.166 | 0.465 | -0.000 | NA | -0.166 |
| 8. Point Judith, RI | NA | NA | -0.132 | NA | NA | -0.054 | -0.379 | NA | 0.052 | -0.029 | NA |
| 9. Clinton, CT | -0.053 | NA | -0.047 | NA | -0.056 | 0.022 | 0.403 | 0.098 | -0.081 | -0.053 | -0.026 |
| 13. Cheesequake, NJ | -0.088 | NA | -0.118 | -0.014 | -0.086 | **-0.438*** | 0.139 | 0.205 | -0.219 | **0.659*** | 0.063 |
| 15. Belford, NJ | **0.510*** | **0.662** | **0.466*** | -0.044 | -0.153 | 0.091 | **0.469*** | 0.142 | **0.406*** | **0.597*** | 0.226 |
| 16. SandyHook, NJ | 0.010 | **0.662** | 0.101 | -0.032 | -0.181 | -0.121 | 0.117 | 0.043 | -0.037 | **0.404** | 0.165 |
| 17. Navesink, NJ | 0.268 | NA | 0.442 | NA | 0.294 | -0.043 | 0.111 | -0.043 | -0.200 | **0.662** | 0.268 |
| 19. Metedeconk, NJ | 0.085 | -0.032 | 0.227 | **0.793*** | -0.063 | -0.145 | **0.466*** | **0.311** | 0.020 | **0.547*** | **0.299** |
| 20. Laurel, NJ | **0.343** | 0.035 | **0.453*** | 0.082 | -0.190 | 0.260 | **0.473*** | **0.358** | 0.177 | **0.613*** | 0.154 |
| 21. Tuckerton, NJ | 0.151 | -0.056 | 0.254 | -0.056 | 0.163 | 0.146 | **0.673** | **0.518** | 0.073 | -0.059 | 0.207 |
| 22. RUMFS, NJ | **0.466*** | -0.077 | 0.280 | 0.487 | **0.365** | -0.073 | **0.280** | 0.081 | 0.043 | **0.814*** | -0.001 |
| 28. Magotha, VA | 0.224 | -0.280 | **0.508** | 0.146 | 0.247 | 0.384 | NA | **1.000** | -0.188 | -0.086 | -0.059 |
| 30. Manteo, NC | -0.100 | -0.280 | -0.261 | -0.226 | -0.307 | -0.166 | -0.097 | **1.000*** | 0.159 | -0.027 | NA |
| 35. Sapelo Island, GA | **0.571** | NA | -0.127 | **0.465** | 0.068 | -0.016 | **1.000** | 0.480 | 0.283 | NA | -0.053 |

|  | **Locus Number** | | | | | | | | |
| --- | --- | --- | --- | --- | --- | --- | --- | --- | --- |
| **Location** | 14 | 15 | 19 | 20 | 22 | 23 | 25 | 26 | 29 |
| 2. Wiscasset, ME | -0.018 | NA | -0.018 | -0.018 | NA | NA | NA | NA | -0.057 |
| 6. Sandwich, MA | -0.378 | NA | NA | -0.056 | -0.188 | -0.086 | NA | -0.118 | -0.188 |
| 8. Point Judith, RI | -0.240 | NA | -0.053 | 0.216 | -0.188 | 0.405 | 0.162 | **0.639** | 0.397 |
| 9. Clinton, CT | NA | NA | -0.081 | 0.098 | -0.026 | -0.081 | -0.026 | 0.341 | 0.034 |
| 13. Cheesequake, NJ | 0.089 | -0.104 | 0.242 | **0.480*** | 0.078 | 0.118 | 0.209 | 0.040 | **0.689*** |
| 15. Belford, NJ | **-0.290** | 0.078 | 0.226 | 0.167 | 0.087 | 0.147 | 0.207 | 0.117 | **0.655*** |
| 16. SandyHook, NJ | **0.365** | 0.242 | **0.530*** | 0.267 | 0.030 | -0.137 | -0.032 | -0.190 | **0.878*** |
| 17. Navesink, NJ | 0.111 | -0.043 | 0.100 | 0.385 | 0.111 | **-0.714** | -0.333 | -0.294 | 0.446 |
| 19. Metedeconk, NJ | 0.077 | 0.147 | **0.481*** | **0.309** | **-0.313** | 0.031 | 0.052 | -0.219 | **0.668*** |
| 20. Laurel, NJ | 0.203 | 0.201 | **0.873*** | **0.418** | 0.177 | -0.052 | 0.191 | -0.041 | 0.291 |
| 21. Tuckerton, NJ | -0.357 | 0.122 | 0.227 | **0.624** | 0.227 | -0.000 | -0.180 | 0.066 | -0.152 |
| 22. RUMFS, NJ | -0.087 | 0.030 | **0.351** | 0.222 | -0.095 | **0.349** | **0.299** | -0.081 | **0.511** |
| 28. Magotha, VA | -0.267 | 0.105 | NA | **0.832** | 0.500 | 0.307 | -0.310 | 0.240 | -0.241 |
| 30. Manteo, NC | -0.086 | **-0.491** | NA | **0.587** | 0.151 | -0.188 | -0.216 | -0.161 | 0.654 |
| 35. Sapelo Island, GA | -0.049 | -0.049 | **0.532** | **0.532** | -0.103 | -0.067 | 0.200 | NA | NA |

**TableS6. 4 cline shape parameters as predicted by ClineFit, arranged by order of increasing centre (as in Fig 3).** Values in brackets represent 2-unit support limits. Markers in red are those exhibiting significant heterozygote deficit at two or more locations; those in blue exhibit significant cytonuclear disequilibrium with one or both of the mitochondrial SNPs; * indicates loci with width less than neutral prediction (614km); ^§^ indicates SNPs with 2-unit support limits of their centre within 10 km of two-unit support limits of mtDNA centres. T is the calculated number of generations since secondary contact, assuming a neutral cline (see equation 1 in the main text)

| Locus Name | Width (km) | Centre (km) | pmin | pmax | T |
| --- | --- | --- | --- | --- | --- |
| 1. Atrial Naturetic Peptide | 934.20 (473.95, 1772.92) | 783.56 (453.75, 986.05) | 0.14 (0.00, 0.31) | 0.89 (0.82, 0.97) | 34725 |
| 2. Chymotrypsinogen | 608.09 (482.21, 763.74) | 837.38 (740.31, 913.86) | 0.01 (0.00, 0.10) | 1.00 (0.98, 1.00) | 14713 |
| 3. Ribosomal Protein S2 | 1681.58 (854.98, 2179.24) | 954.38 (752.26, 1121.91) | 0.00 (0.00, 0.17) | 1.00 (0.83, 1.00) | 112511 |
| 4. Translationally Controlled Tumor Protein | 526.69 (265.31, 876.49) | 993.59 (836.94, 1108.65) | 0.44 (0.29, 0.57) | 0.98 (0.92, 1.00) | 11037 |
| 5. Ribosomal protein | 627.07 (322.39, 1015.45) | 1024.05 (877.03, 1142.12) | 0.16 (0.06, 0.32) | 0.93 (0.87, 0.98) | 15646 |
| 6. Guanine nucleotide binding protein (RACK1) | 622.71 (486.43, 847.31) | 1087.80 (1015.94, 1131.71) | 0.00 (0.00, 0.00) | 0.93 (0.87, 0.98) | 15429 |
| 7. NACA | 752.47 (295.31, 1096.48) | 1087.95 (966.98, 1192.90) | 0.15 (0.05, 0.30) | 1.00 (0.94, 1.00) | 22529 |
| 8. Titin Cap | 900.33 (234.08, 1298.01) | 1141.10 (1064.77, 1248.39) | 0.00 (0.00, 0.16) | 0.90 (0.80, 0.99) | 32253 |
| 9. 1176* | 305.53 (122.42, 550.46) | 1182.75 (1114.94, 1232.99) | 0.01 (0.00, 0.05) | 0.59 (0.48, 0.70) | 3714 |
| 10. 60S ribosomal protein L6 | 418.27 (259.25, 658.27) | 1191.22 (1137.27, 1231.96) | 0.05 (0.01, 0.12) | 0.86 (0.77, 0.94) | 6961 |
| 11. Parvalbumin ^δ^ | 313.67 (165.73, 781.46) | 1238.19 (1165.99, 1293.13) | 0.18 (0.10, 0.26) | 0.73 (0.64, 0.83) | 3915 |
| 12. Actin binding LIM protein family 3*^δ^ | 117.61 (88.33, 153.91) | 1256.71 (1245.24, 1267.36) | 0.00 (0.00, 0.03) | 0.87 (0.80, 0.92) | 550 |
| 13. Myoglobin* ^δ^ | 258.80 (181.19, 532.76) | 1261.74 (1231.34, 1287.52) | 0.06 (0.00, 0.13) | 0.85 (0.77, 0.91) | 2665 |
| 14. 60Sribosomal protein L35^§^ | 204.85 (113.87, 1547.02) | 1262.23 (1223.82, 1494.24) | 0.12 (0.01, 0.20) | 0.66 (0.57, 1.00) | 1670 |
| 15. 40S ribosomal protein S17* ^δ^ | 140.71 (93.61, 205.54) | 1265.91 (1247.06, 1282.56) | 0.24 (0.16, 0.33) | 0.97 (0.93, 0.99) | 788 |
| 16. Cytochrome B* | 75.16 (56.31, 93.84) | 1267.09 (1258.56, 1274.24) | 0.00 (0.00, 0.01) | 0.96 (0.90, 0.99) | 225 |
| 17. Cytochrome c oxidase subunit I* | 83.78 (70.41, 98.93) | 1266.82 (1260.31, 1273.55) | 0.00 (0.00, 0.02) | 1.00 (0.98, 1.00) | 279 |
| 18. Phosphate carrier protein* ^δ^ | 184.11 (93.27, 367.30) | 1303.14 (1281.50, 1344.20) | 0.08 (0.03, 0.15) | 0.78 (0.69, 0.86) | 1349 |
| 19. HD domain containing 2 (HDDC2) * ^δ^ | 260.32 (165.00, 390.74) | 1306.84 (1284.68, 1337.72) | 0.06 (0.02, 0.13) | 0.96 (0.90, 0.99) | 2696 |
| 20. Warm acclimation related protein* | 173.65 (94.07, 419.00) | 1319.63 (1296.15, 1382.30) | 0.15 (0.10, 0.22) | 0.82 (0.74, 0.91) | 1200 |
| 21. Glyceraldehyde 3 phosphate dehydrogenase* | 279.99 (136.68, 451.49) | 1326.38 (1287.44, 1378.44) | 0.39 (0.30, 0.49) | 0.99 (0.95, 1.00) | 3119 |
| 22. Tropomyosin* | 264.42 (134.79, 476.09) | 1326.79 (1295.54, 1375.65) | 0.14 (0.07, 0.23) | 0.85 (0.76, 0.92) | 2782 |
| 23. Lactate dehydrogenase B654 | 561.49 (282.65, 802.19) | 1360.23 (1298.24, 1417.55) | 0.12 (0.05, 0.21) | 0.99 (0.88, 1.00) | 12544 |
| 24. Chymotrypsin-C* | 150.05 (0.10, 551.90) | 1382.38 (1326.52, 1499.86) | 0.17 (0.07, 0.22) | 0.64 (0.55, 0.75) | 896 |
| 25. Nucleotide diphosphate kinase 1 | 467.51 (331.31, 659.73) | 1395.28 (1345.25, 1449.92) | 0.18 (0.11, 0.28) | 1.00 (0.95, 1.00) | 8696 |
| 26. Lactate dehydrogenase B1033 | 931.92 (696.26, 1201.27) | 1399.80 (1339.45, 1458.73) | 0.00 (0.00, 0.07) | 1.00 (0.90, 1.00) | 34556 |
| 27. Hemoglobin alpha* | 341.43 (241.08, 441.31) | 1445.74 (1386.30, 1487.74) | 0.10 (0.06, 0.15) | 1.00 (0.92, 1.00) | 4638 |
| 28. Cytochrome p450 | 591.49 (355.06, 954.05) | 1474.93 (1395.25, 1584.35) | 0.08 (0.02, 0.17) | 0.91 (0.77, 1.00) | 13921 |
| 29. 14-3-3 protein zeta | 1076.98 (645.76, 1367.98) | 1482.38 (1337.63, 1556.16) | 0.00 (0.00, 0.06) | 1.00 (0.77, 1.00) | 46150 |
| 30. Hemoglobin beta* | 457.40, (363.90, 580.64) | 1518.09 (1473.77, 1565.98) | 0.03 (0.01, 0.07) | 1.00 (0.93, 1.00) | 8324 |
| 31. Nucleotide diphosphate kinase 2 | 406.89 (43.61, 764.39) | 1536.48 (1437.40, 1647.93) | 0.17 (0.09, 0.24) | 0.90 (0.72, 1.00) | 6587 |
| 32. 1173 ^δ^ | 1749.25 (815.18, 3100.53) | 1590.03 (1265.50, 1798.30) | 0.08 (0.00, 0.25) | 1.00 (0.71, 1.00) | 121749 |
